# Recent Progress in Spinel Ferrite (MFe_2_O_4_) Chemiresistive Based Gas Sensors

**DOI:** 10.3390/nano13152188

**Published:** 2023-07-27

**Authors:** Run Zhang, Cong Qin, Hari Bala, Yan Wang, Jianliang Cao

**Affiliations:** 1 School of Materials Science and Engineering, Henan Polytechnic University, Jiaozuo 454000, China; zhangrun0518@home.hpu.edu.cn (R.Z.); hari@hpu.edu.cn (H.B.); 2 College of Chemistry and Chemical Engineering, Henan Polytechnic University, Jiaozuo 454000, China; qincong@hpu.edu.cn; 3 College of Safety Science and Engineering, Henan Polytechnic University, Jiaozuo 454003, China; 4 State Collaborative Innovation Center of Coal Work Safety and Clean-Efficiency Utilization, Henan Polytechnic University, Jiaozuo 454003, China

**Keywords:** spinel ferrite, metal oxide semiconductor, chemiresistive gas sensor, nanostructure, doping, heterostructure

## Abstract

Gas-sensing technology has gained significant attention in recent years due to the increasing concern for environmental safety and human health caused by reactive gases. In particular, spinel ferrite (MFe_2_O_4_), a metal oxide semiconductor with a spinel structure, has emerged as a promising material for gas-sensing applications. This review article aims to provide an overview of the latest developments in spinel-ferrite-based gas sensors. It begins by discussing the gas-sensing mechanism of spinel ferrite sensors, which involves the interaction between the target gas molecules and the surface of the sensor material. The unique properties of spinel ferrite, such as its high surface area, tunable bandgap, and excellent stability, contribute to its gas-sensing capabilities. The article then delves into recent advancements in gas sensors based on spinel ferrite, focusing on various aspects such as microstructures, element doping, and heterostructure materials. The microstructure of spinel ferrite can be tailored to enhance the gas-sensing performance by controlling factors such as the grain size, porosity, and surface area. Element doping, such as incorporating transition metal ions, can further enhance the gas-sensing properties by modifying the electronic structure and surface chemistry of the sensor material. Additionally, the integration of spinel ferrite with other semiconductors in heterostructure configurations has shown potential for improving the selectivity and overall sensing performance. Furthermore, the article suggests that the combination of spinel ferrite and semiconductors can enhance the selectivity, stability, and sensing performance of gas sensors at room or low temperatures. This is particularly important for practical applications where real-time and accurate gas detection is crucial. In conclusion, this review highlights the potential of spinel-ferrite-based gas sensors and provides insights into the latest advancements in this field. The combination of spinel ferrite with other materials and the optimization of sensor parameters offer opportunities for the development of highly efficient and reliable gas-sensing devices for early detection and warning systems.

## 1. Introduction

Metal oxide semiconductor (MOS) gas sensors operate by detecting alterations in the electrical conductivity of a semiconducting metal oxide when exposed to a gas [1]. When the MOS sensor comes into contact with the target gas, the gas molecules adhere to the sensor material’s surface, resulting in a modification of the sensor’s electrical resistance [2]. The extent and direction of the resistance alteration correlate with the gas concentration and its chemical properties. Numerous metal oxide semiconducting materials, such as tin oxide (SnO_2_) [3], zinc oxide (ZnO) [4], titanium dioxide (TiO_2_) [5], and tungsten oxide (WO_3_) [6], have been widely utilized in the production of MOS sensors. These materials exhibit diverse sensing characteristics towards various gases, and their sensitivity, selectivity, and stability can be adjusted via material doping, surface modification, and operating conditions. To enhance the performance of MOS gas sensors, novel sensing structures such as nanowires [7], nanotubes [8], and nanostructured thin films [9] have been developed, offering larger surface-to-volume ratios and improved gas adsorption capabilities. Additionally, advanced fabrication techniques such as atomic layer deposition (ALD) [10], chemical vapor deposition (CVD) [11], and spray pyrolysis [12] have been employed to achieve precise control over the sensor’s morphology, composition, and functionality. In conclusion, MOS gas sensors have become indispensable tools for monitoring environmental pollution, ensuring industrial safety, and safeguarding public health [13,14]. The ongoing progress in sensor technology and its integration with information and communication systems will create novel opportunities for real-time, reliable, and intelligent gas-sensing solutions.

MOS-gas-sensitive materials can be classified into two categories based on the number of metal ions present in the single-phase metal oxide material: single metal oxides and composite metal oxides. The gas sensors based on single metal oxides exhibit excellent attributes, including easy integration, good repeatability, and effective detection of various gases [15,16,17]. Nonetheless, there is still room for improvement in terms of the selectivity and recovery performance of single-phase gas-sensitive materials. Researchers have explored strategies to enhance the sensing performance by incorporating precious metal catalysts or combining them with other materials to modify the morphology of single metal oxides, aiming to provide activation energy for reactions or form p–n heterojunctions.

In recent times, the distinctive magnetic properties [18], electrical properties [19], microwave absorption [20], and photocatalytic properties [21] of composite metal oxides, specifically spinel ferrites, have garnered significant attention. The primary preparation techniques for MFe_2_O_4_-based gas-sensitive materials include the co-precipitation method [22,23,24,25], sol–gel method [26,27,28], and template synthesis method [29]. These methods enable the production of spinel ferrite nanomaterials with diverse morphologies such as nanorods, nanotubes, nanofilms, and core–shell microspheres. The combination of novel synthesis approaches and the integration of new functional materials has led to the development of spinel ferrite and spinel ferrite composite materials with controllable structures and morphologies, thereby expanding their application potential. For instance, the controlled synthesis of spinel ferrite nanoparticles has exhibited promising outcomes in biomedical applications such as drug delivery and cancer therapy [30]. Furthermore, the combination of spinel ferrite with graphene oxide enhances its magnetic and electrical properties, positioning it as a potential candidate for spintronics [31] and electromagnetic shielding applications [32]. Additionally, the incorporation of metal ions or other functional materials into spinel ferrite has shown improved catalytic and photocatalytic properties, thereby finding application in areas such as wastewater treatment [33] and hydrogen production [34]. Overall, the advancement of novel synthesis methods and the integration of functional materials have broadened the scope of zinc ferrite materials and opened up avenues for future research.

As a semiconducting, magnetic oxide material, spinel ferrite has excellent chemical stability, enabling the effective adsorption of various gases [35]. Its inherent catalytic properties stimulate chemisorption processes that result in changes in its electrical resistance when exposed to different gases [36]. This allows for accurate gas detection and measurement. Additionally, spinel ferrite can operate at lower temperatures compared with other gas sensors, which leads to increased energy efficiency [37]. Its high sensitivity [38] and selectivity [39] towards particular gases, coupled with its capacity for miniaturization, make spinel ferrite an optimal material for building reliable, efficient, and compact gas sensors.

This review article is organized as follows: Section 2 presents an introduction to the gas-sensing mechanism of spinel ferrite. Section 3, Section 4 and Section 5 present a detailed review of the recent advancements in spinel-ferrite-based gas-sensing materials for the detection of reducing gases, categorized based on the types of gas-sensing enhancement mechanisms. Finally, in Section 6, a summary and outlook for this review are provided, emphasizing the potential future directions for spinel-ferrite-based gas-sensing materials and their applications.

## 2. Gas-Sensing Mechanism

**Figure 1:**
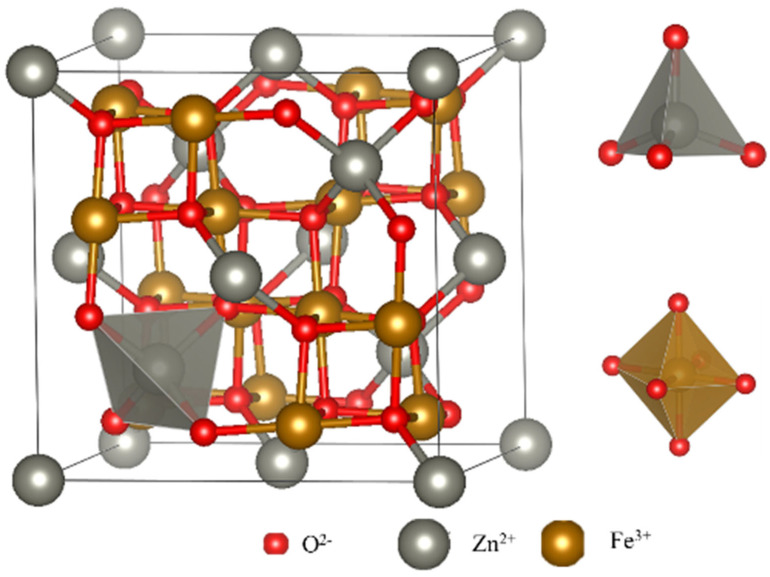
The crystal structure of ZnFe_2_O_4_, with Zn^2+^ in the tetrahedron gap and Fe^3+^ in the octahedron gap.

With its spinel crystal structure, spinel ferrite emerges as a promising sensing material possessing exceptional properties. Figure 1 illustrates the crystal structure of zinc ferrite, where the face-centered cube of O^2−^ accumulates within its crystal lattice, while the metal ions M^2+^ and Fe^3+^ are embedded in the tetrahedral and octahedral gaps formed by O^2−^. This structure readily facilitates the formation of defects, including oxygen vacancies, both internally and on the surface, making it highly advantageous for gas-sensitive materials. The unique crystal structure, specifically the insertion of the transition metal cation Zn^2+^ into the Fe^2+^Fe^3+^O_4_ structure, plays a crucial role in the effective detection of reducing gases.

The gas-sensing response of spinel ferrite is determined by the complex interaction that occurs at the interface between the gas and solid material. However, a unified definition of gas sensor mechanisms is lacking. A commonly proposed sensing mechanism for spinel ferrite sensors is as follows: when a spinel-ferrite-based sensor is exposed to air, oxygen molecules adsorb onto its surface, capturing free electrons from the conduction band and forming oxygen anions. The specific form of these oxygen anions depends on the operating temperature. The loss of electrons generates an electron depletion layer (n-type) on the semiconductor surface, resulting in an increase in resistance. In a reducing gas atmosphere, Equation (6) occurs, leading to a reduction in the resistance of the electron depletion region and sensor. It is worth noting that the reaction described in Equation (6) may vary depending on the operating temperature or target gas.

(1) $$O_{2}\left(\mathrm{gas}\right)\to O_{2}\left(\mathrm{ads}\right)$$

(2) $$O_{2}\left(\mathrm{ads}\right)+e^{-}\to O_{2}^{-}\left(\mathrm{ads}\right)\;\;\;\;T<150{\;}^{\circ }C$$

(3) $$O_{2}^{-}\left(\mathrm{ads}\right)+e^{-}\to O^{2-}\left(\mathrm{ads}\right)\;\;\;150^{\circ }C<T<400^{\;\circ }C$$

(4) $$O^{-}\left(\mathrm{ads}\right)+e^{-}\to O^{2-}\left(\mathrm{ads}\right)\;\;\;\;T>400{\;}^{\circ }C$$

(5) $$G\left(\mathrm{gas}\right)\to G\left(\mathrm{ads}\right)$$

(6) $$G\left(\mathrm{ads}\right)+O^{-}\to \mathrm{GO}+e^{-}\;\;\;150^{\circ }C<T<400^{\;\circ }C$$

The unique microstructure and high specific surface area of pure MFe_2_O_4_ nanomaterials offer numerous adsorption sites, leading to an enhancement in gas-sensing performance. The addition of metal ions through doping reduces the barrier height of grain boundaries, facilitating improved carrier diffusion and transfer rates; heterostructures [40], on the other hand, allow for the modulation of the electron depletion region and potential barrier at the interface by leveraging the interaction between Fermi energy levels and energy bands [41]. These mechanisms collectively contribute to the enhancement of gas sensitivity in the respective materials. More detailed explanations of the gas-sensitive mechanisms specific to these new materials can be found in Section 3 (Nanostructures), Section 4 (Doping), and Section 5 (Heterostructures).

## 3. Nanostructure

The gas-sensing application has significantly benefited from the use of nanostructured materials, primarily due to their high surface-to-volume ratio, which allows for better interaction with the gas molecules. In particular, zinc ferrite, a type of spinel ferrite, has been widely used due to its specific surface area, contact area, porosity, grain size, and grain stacking order. These factors all contribute to its gas-sensing properties. The operating temperature, humidity, and gas concentration are several external factors that can influence the performance of zinc ferrite-based gas sensors. For instance, at higher operating temperatures, the sensor’s sensitivity can increase due to the enhanced surface reaction rates [42]. On the other hand, excessive humidity may cause the surface of the sensor to become water-saturated, which could inhibit its response to target gases [35]. Apart from these external factors, the morphology-related characteristics of spinel ferrite also play a significant role in its gas-sensing properties. The development of unique morphologies and structures in spinel ferrite is considered a promising approach to enhance its gas-sensing performance. For example, porous spinel ferrite with large specific surface areas can provide more active sites for gas molecule adsorption, facilitating improved surface effects, electronic transfer efficiency, and ultimately a better gas-sensing performance. Various synthesis methods can be employed to create spinel ferrite materials with different morphologies. These include sol–gel, hydrothermal, and co-precipitation methods, among others. Each method offers unique advantages in terms of controlling the size, shape, and distribution of the nanoparticles, thereby allowing for the optimization of the sensor’s performance. In the subsequent sections, we will delve deeper into these topics, providing a comprehensive review of the latest research findings on ferrite sensors with diverse nanostructures. We will also discuss the special properties of these sensors as documented in existing literature (Table 1, Table 2, Table 3 and Table 4). We believe that this review will provide valuable insights into the ongoing advancements in the field of spinel-ferrite-based gas sensors and highlight potential avenues for future research.

### 3.1. Nanoparticles

The preparation method for spinel ferrite nanoparticles can be achieved through the following steps: First, an appropriate synthesis method, such as sol–gel [109], hydrothermal [101], or co-precipitation [90], is used to mix suitable metal salts with basic precipitants, forming a precipitate. Next, through appropriate washing, centrifugation, and drying processes, the precipitate is transformed into nanoparticle form. Finally, through heat treatment or other surface modification methods, the morphology and properties of the nanoparticles can be controlled [111]. The size of nanoparticles and nanocrystals is not primarily dependent on the synthesis method employed, but rather, it is mainly influenced by the preparation and control of the salt solution.

**Figure 2:**
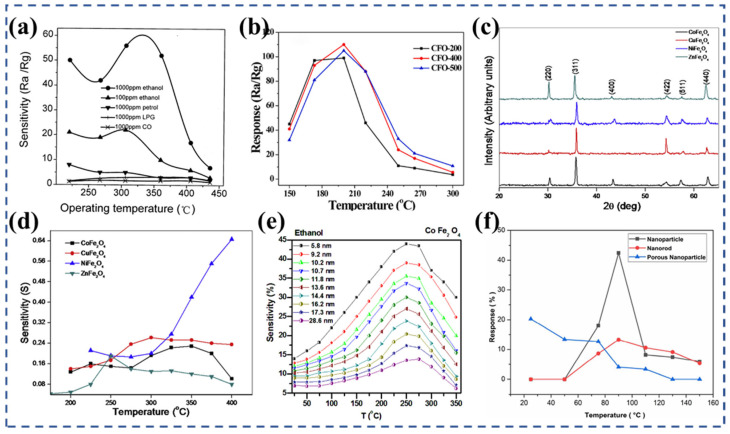
(**a**) The effect of operating temperature of the CdFe_2_O_4_ sensor on the various gas responses. (**b**) Response of the sensors to 100 ppm ethanol at different operating temperatures. (**c**) XRD pattern of (Co, Cu, Ni, and Zn) ferrite thin films. (**d**) Response value of (Co, Cu, Ni, and Zn) ferrite thin films to 5 ppm LPG at different operating temperatures. (**e**) Size of CoFe_2_O_4_ nanoparticles dependent on response value (%) with varying temperatures for 200 ppm ethanol. (**f**) The sensitivity of individual CoFe_2_O_4_ sensors to 100 ppm methanol across varying temperature conditions. (**a**) Reproduced with permission [43], copyright 1998, Elsevier B.V. (**b**) Reproduced with permission [35], copyright 2022, Elsevier B.V. (**c**,**d**) Reproduced with permission [51], copyright 2015, Elsevier B.V. (**e**) Reproduced with permission [52], copyright 2015, IEEE Xplore. (**f**) Reproduced with permission [54], copyright 2020, IEEE Xplore.

By dispersing pure CdSO_4_·8/3H_2_O and Fe(NO_3_)_3_·9H_2_O in ultra-pure water, Liu et al. [43] prepared mixed salt solutions with different Cd/Fe molar ratios, combined with co-precipitation and calcination at different temperatures to prepare CdO-Fe_2_O_3_ composite oxide particles. According to XRD verification, the sample with a Cd/Fe ratio of 1/2 was identified as a spinel phase CdFe_2_O_4_, which exhibited the highest sensitivity (48) towards ethanol at 300 °C (Figure 2a). The study conducted by Rao et al. [51] focused on the utilization of the spray pyrolysis deposition technique to fabricate nanocrystalline (Co, Cu, Ni, and Zn) ferrite thin film sensors. The XRD patterns (Figure 2c) show the single cubic spinel phase of the (Co, Cu, Ni, and Zn) ferrite. From Figure 2d, the sensing characteristics of these sensors indicate that the ZnFe_2_O_4_ nanocrystalline is more suitable as a sensor at lower temperatures and concentrations. On the other hand, the NiFe_2_O_4_ nanocrystalline demonstrates an outstanding LPG sensing ability at higher temperatures.

As discussed earlier, optimizing the grain size and specific surface area of spinel ferrite can significantly enhance the performance of gas sensors. Wei et al. [35] prepared CoFe_2_O_4_ nanoparticles via a hydrothermal method. The CFO-400 sensor, which is calcined at 400 °C, shows promising results with its response value of 110 to 100 ppm ethanol gas at 200 °C (Figure 2b). This not only indicates its high sensitivity, but also showcases its good repeatability and stability, which are crucial characteristics for sensor materials. Rathore et al. [52] prepared CoFe_2_O_4_ nanoparticles with varying particle sizes through the uniaxial press method. The objective of the research was to examine how the sensing performance of the nanoparticles is influenced by factors such as particle size, temperature, and gas flow. The results of the study demonstrated that CoFe_2_O_4_ nanoparticles have good gas sensitivity, and the maximum response value increases with the decrease in particle size. Among them, the response value of 5.8 nm CoFe_2_O_4_ nanoparticles to 5 ppm LPG at 250 °C is the highest, reaching 0.72 (Figure 2e), and its response time and recovery time are 3 s and 48 s, respectively. Halvaee et al. [54] employed a hydrothermal synthesis technique to fabricate three distinct nanostructures of CoFe_2_O_4_, namely nanoparticles, nanorods, and porous nanoparticles. The structures and properties of these nanostructures were analyzed. A cost-effective gas sensor, constructed using a printed circuit board, was utilized to measure methanol gas and assess its performance at different temperatures (Figure 2f). The optimal operating temperatures for the three sensors were found to be 90 °C and room temperature, respectively. At 90 °C, the CoFe_2_O_4_ nanoparticles exhibited a maximum response value of 42.4%, while the CoFe_2_O_4_ porous nanoparticles demonstrated a maximum response value of 20.26% at room temperature. The CoFe_2_O_4_ nanorods, on the other hand, displayed a maximum response value of 13.3% at 90 °C. In the porous nanoparticle sensor, the optimal temperature was reduced to room temperature due to the high surface volume ratio of the structure.

**Figure 3:**
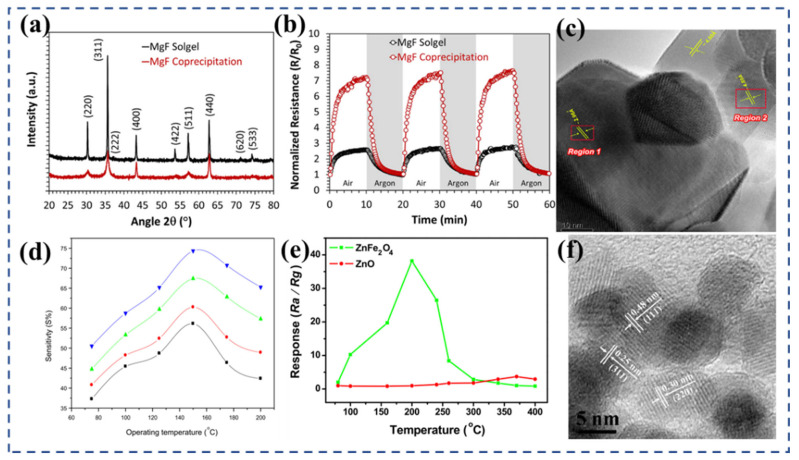
(**a**) XRD of the synthesized MgFe_2_O_4_ samples. (**b**)Variation in the response of MgFe_2_O_4_ samples at 300 °C. (**c**) HRTEM image showing cubic NiFe_2_O_4_. (**d**) H_2_S sensitivity of NiFe_2_O_4_ with various milled times at operating temperatures. (**e**) Responses of sensors to 500 ppm acetone at various temperatures. (**f**) TEM images of the synthesized ZnFe_2_O_4_ nanoparticles. (**a**,**b**) Reproduced with permission [69], copyright 2018, Elsevier B.V. (**c**,**d**) Reproduced with permission [78], copyright 2015, Elsevier B.V. (**e**,**f**) Reproduced with permission [92], copyright 2015, Elsevier B.V.

Sumangala et al. [69] synthesized the MgFe_2_O_4_ nanoparticles employing both the co-precipitation and sol–gel methods. The XRD patterns presented in Figure 3a demonstrate the similarity in the structural characteristics of both samples. The co-precipitation sample exhibited a smaller particle size and twice the BET surface area compared with the sol–gel combustion sample. The electrical properties and CO_2_ sensing capabilities of these two MgFe_2_O_4_ nanoparticles were investigated (Figure 3b). Notably, the co-precipitated sample demonstrated a higher sensing response of 36%, whereas the sol–gel combusted sample achieved a sensing response of 24%. Ghosh et al. [78] reported nanocrystalline NiFe_2_O_4_ (Figure 3c) through the sol–gel auto-combustion method. Ball milling was performed at room temperature and particle size was controlled to optimize the sensitivity of H_2_ and H_2_S. The experimental results show that there was a notable enhancement in the gas response when the particle size was reduced or the specific surface area was increased (Figure 3d). Compared with the other test gases, NiFe_2_O_4_ nanocrystals with a particle size of ~5.35 nm had a response value of ~58% to 200 ppm H_2_ at 100 °C and ~75% to 200 ppm H_2_S at 150 °C.

In a study conducted by Karpova et al. [88], ZnO, Fe_2_O_3_, and zinc ferrite ZnFe_2_O_4_ nanopowders were prepared using the co-precipitation method. The gas-sensitive results proved that the sensitivity of ZnFe_2_O_4_ towards ethanol and acetone was significantly higher compared with the simple oxides, with values ranging from one to two orders of magnitude greater, respectively. This enhanced gas sensitivity of ZnFe_2_O_4_ can be attributed to the presence of a high concentration of acidic Bronsted centers that contain active protons. These centers facilitate participation in REDOX reactions and selectively adsorb ethanol based on the acid−base mechanism. Using the hydrothermal method, Zhang et al. [92] successfully synthesized ZnFe_2_O_4_ nanoparticles (about 10 nm) (Figure 3f). The phase and morphology of the prepared products were strongly influenced by the reaction conditions, including the reaction time, temperature, and the molar ratio of raw materials. The experimental findings (Figure 3e) revealed that the prepared ZnFe_2_O_4_ nanoparticles exhibited a significantly higher response value of 39.5 to 200 ppm acetone compared with the precursor ZnO, which only had a response value of 4.2, at 200 °C.

**Figure 4:**
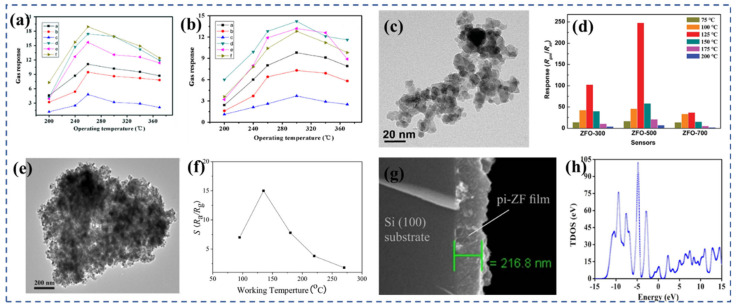
(**a**) The sensitivity–temperature characteristics of various MFe_2_O_4_ sensors in detecting formaldehyde. (**b**) The sensitivity-temperature characteristics of various MFe_2_O_4_ sensors in detecting formaldehyde ethanol: (**a**) Fe_3_O_4_, (**b**) CoFe_2_O_4_, (**c**) NiFe_2_O_4_, (**d**) MgFe_2_O_4_, (**e**) CdFe_2_O_4_, (**f**) ZnFe_2_O_4_. (**c**) TEM image of the ZnFe_2_O_4_ nanoparticles. (**d**) Comparative analysis of the NO_2_ response among sensors based on ZFO-300, ZFO-500, and ZFO-700 materials when exposed to 10 ppm NO_2_ at varying operating temperatures. (**e**) The TEM image of ZnFe_2_O_4_ nanoparticles at low magnification. (**f**) The response values of sensors based on ZnFe_2_O_4_ nanoparticles to 5 ppm H_2_S gas at different working temperatures. (**g**) Cross-sectional FESEM image of the ZnFe_2_O_4_ film. (**h**) Total density of states (TDOS) of the ZnFe_2_O_4_. (**a**,**b**) Reproduced with permission [93], copyright 2016, Elsevier B.V. (**c**,**d**) Reproduced with permission [99], copyright 2019, Royal Society of Chemistry. (**e**,**f**) Reproduced with permission [110], copyright 2018, Elsevier B.V. (**g**,**h**) Reproduced with permission [102], copyright 2022, IEEE Xplore.

Cao et al. [93] employed a solid-state chemical reaction to synthesize various MFe_2_O_4_ (M = Fe, Co, Ni, Mg, Cd, and Zn) ferrite materials with distinct morphologies. Compared with traditional semiconductor oxides, these prepared ferrites exhibited enhanced gas sensitivity at lower operating temperatures and demonstrated rapid response and recovery characteristics. At 260 °C, ZnFe_2_O_4_ displayed a response value of 37.3 towards 100 ppm methanol (Figure 4a), which was the highest gas response among the different ferrites. It exhibited a response value of 29.1 towards 100 ppm ethanol (Figure 4b) withfast r esponse and recovery times of 5 s and 26 s, respectively. Li et al. [99] successfully synthesized ultra-small ZnFe_2_O_4_ nanoparticles (Figure 4c) using the hydrothermal synthesis method. These nanoparticles exhibited excellent selectivity towards NO_2_ molecules. The ZnFe_2_O_4_-based sensor showed an impressive response with a gas-to-air ratio (R_gas_/R_air_) of 247.7 toward 10 ppm NO_2_ at 125 °C (Figure 4d), which is a relative low temperature. It also demonstrated a fast response and recovery characteristic (6.5 s/11 s). Li et al. [94] further investigated the mechanism behind the superior selectivity and sensing performance of ZnFe_2_O_4_ towards NO_2_ compared with other gases. Through non-in situ photoluminescence (PL) characterization and density functional theory (DFT) calculations, they found that the gas-sensitive mechanism of ZnFe_2_O_4_ towards NO_2_ is based on surface charge transfer. The presence of oxygen vacancies in the material also enhanced the adsorption energy and charge transfer between ZnFe_2_O_4_ and NO_2_ molecules on the surface.

Zhang et al. [110] synthesized ZnFe_2_O_4_ nanoparticles using a solvothermal method with zinc acetylacetone and iron acetylacetone as the precursors. By carrying out the synthesis at 150 °C, ZnFe_2_O_4_ nanoparticles (Figure 4e) with a diameter of approximately 20 nm were obtained. These ZnFe_2_O_4_ nanoparticles exhibited excellent gas-sensing capabilities, particularly for H_2_S gas. The sensor was able to detect H_2_S gas as low as 1 ppm at a temperature of 135 °C, with a sensor response reaching 15.1 for 5 ppm H_2_S gas at the same temperature (Figure 4f). These results suggest that nano-ZnFe_2_O_4_ holds great promise for the development of H_2_S gas sensors. The group of Jha et al. [102] conducted a study on a selective hydrogen H_2_S gas sensor based on a zinc ferrite film (Figure 4g). The film was prepared using microwave-assisted solvent-thermal deposition. The sensor exhibited an excellent performance at an operating temperature of 250 °C. The response range of the sensor was found to be 1872–90% for H_2_S gas concentrations ranging from 5.6 ppm to 0.3 ppm. Through density functional theory calculations, the researchers concluded that the rapid rise and fall times of H_2_S (approximately 40 s and 70 s, respectively) and the complete recovery of the device were attributed to the physical adsorption of H_2_S molecules on the partially reversed ZnFe_2_O_4_ surface. Figure 4h shows the total density of states (TDOS) of the ZnFe_2_O_4_. In the experiment, a double-difference subtraction automatic balance interface circuit was utilized to drive the sensor, and the noise signal was accurately processed and compensated through the differential output.

### 3.2. Nanorods/Nanotubes

The synthesis methods for spinel ferrite nanorods, nanotubes, and nanowires primarily include hydrothermal [113] and electrospinning [123] techniques. Nanofibers constructed via electrospinning exhibit uniformity and smoothness, thus making the technique widely utilized in the preparation of one-dimensional materials.

In the field of gas sensing, there is a growing interest in one-dimensional (1D) nanostructures such as nanorods, nanotubes, and nanowires, as they are gaining more attention compared with nanoparticles. The reasons for this are manifold. (1) One-dimensional nanostructures often have more active sites compared with nanoparticles. These active sites are the locations where the gas molecules can interact with the material, thereby inducing a detectable change (such as a change in resistance). Therefore, having more active sites means the material can interact with more gas molecules simultaneously, enhancing the sensitivity of the sensor [111]. (2) One-dimensional nanostructures such as nanotubes have unique gas diffusion characteristics. Their channel-like structure allows gas molecules to easily diffuse and permeate through the material. This not only increases the interaction between the gas and the material, but also improves the speed of detection, making the sensor more responsive [119]. (3) Nanotubes and similar structures typically have a relatively high specific surface area [114]. A higher surface area means more space for gas molecules to interact with the material, which further improves the sensitivity of the sensor. One-dimensional nanostructures are known for their favorable electron characteristics. For instance, nanowires can efficiently transport carriers, which is crucial in transducing the interaction between the gas and the material into a detectable electrical signal. In summary, because of their unique structural and electronic properties, materials with 1D nanostructures such as nanorods, nanotubes, and nanowires offer significant advantages in gas sensing and are being actively explored as potential gas-sensing materials.

**Figure 5:**
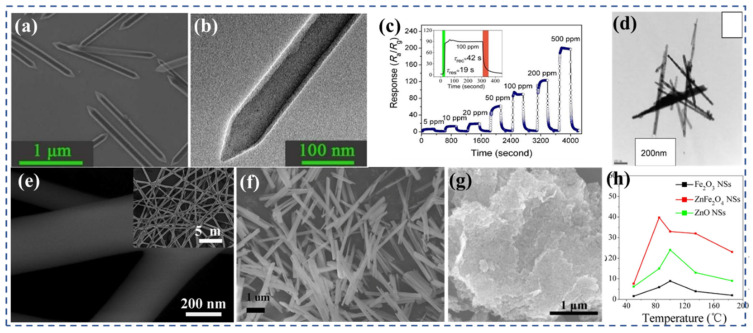
(**a**) SEM image and (**b**) TEM image of as-prepared NiFe_2_O_4_ nanorods. (**c**) The dynamic response−recovery characteristics of NiFe_2_O_4_ nanorods to n-propanol at different concentrations. Insert: response and recovery curve of the sensor to 100 ppm n-propanol. (**d**) TEM image of NiFe_2_O_4_ nanorods. (**e**) SEM images of ZnFe_2_O_4_ nanofiber. (**f**) SEM images of porous ZnFe_2_O_4_ nanorods. (**g**) SEM images of ZnFe_2_O_4_ nanosheets. (**h**) The response values of the sensors to 1 ppm H_2_S at various operating temperatures. (**a**–**c**) Reproduced with permission [115], copyright 2018, Wiley-VCH. (**d**) Reproduced with permission [116], copyright 2007, Elsevier B.V. (**e**) Reproduced with permission [123], copyright 2018, Elsevier B.V. (**f**) Reproduced with permission [122], copyright 2017, Elsevier B.V. (**g**,**h**) Reproduced with permission [129], copyright 2017, Elsevier B.V.

To investigate the impact of structure on the gas-sensing performance of a sensor, Zhang et al. [113] utilized a high-efficiency anodic alumina template method and a hydrothermal method to prepare NiFe_2_O_4_ hollow nanotubes with a length of 1 μm and a diameter of 100 nm, as well as NiFe_2_O_4_ nanoparticles, respectively. In comparison with the NiFe_2_O_4_ nanoparticles sensor, the NiFe_2_O_4_ nanotube sensor possessed a porous structure with overlapping nanotubes, which facilitated improved gas sensitivity. During testing with different NH_3_ gas concentrations, the NiFe_2_O_4_ nanotubes sensor exhibited a higher response compared with the NiFe_2_O_4_ nanoparticles sensor, albeit with a slower recovery speed. The high specific surface area of the nanotubes played a crucial role in the ability of the NiFe_2_O_4_ nanotubes sensor to detect NH_3_ gas. Wang et al. [115] developed a novel gas-sensing material, NiFe_2_O_4_ porous nanorods (Figure 5a,b), which exhibited improved sensitivity and selectivity for detecting the harmful gas n-propanol. These porous javelin-such as nanorods were synthesized using Ni/Fe bimetallic metal–organic frameworks as templates. As a gas-sensing material, ferrite demonstrated n-type gas-sensing behavior with reduced resistance in a reducing gas atmosphere. The NiFe_2_O_4_ nanorods exhibited an outstanding sensing performance for n-propanol (Figure 5c), with an extremely low detection limit of 0.41 ppm at 120 °C. At the same time, the sensor had a good selectivity to n-propanol, good cycle stability, and long-term stability. The exceptional performance of NiFe_2_O_4_ nanorods can be attributed to their distinctive morphology and porous structure. The large number of reaction sites offered by the porous structure facilitated the accelerated diffusion of n-propanol gas, allowing the sensor to quickly and accurately detect the presence of the gas. Chu et al. [116] conducted a study where they prepared NiFe_2_O_4_ nanorods (Figure 5d) and nanocubes using the hydrothermal method. The nanorods had a length of approximately 1 μm and a diameter of about 30 nm, while the nanocubes had a side length of around 60–100 nm. The results of the study showed that the sensor based on NiFe_2_O_4_ nanorods exhibited high sensitivity and selectivity towards triethylamine. Specifically, it achieved a sensitivity of 7 when detecting 1 ppm of triethylamine at 175 °C. However, the NiFe_2_O_4_ nanocube-based sensor demonstrated a unique conductivity response in the NH_3_ environment, showing a significant increase. Specifically, when exposed to 500 ppm triethylamine, the sensor exhibited a response of 0.033. In contrast, the sensors based on NiFe_2_O_4_ nanocubes exhibited a different behavior. In a reducing gas atmosphere, the conductivity of the sensor increased. The shape of the crystal, whether nanorods or nanocubes, significantly influenced not only the response value of the gas, but also the type of semiconductor behavior observed.

Nguyen et al. [123] demonstrated the sensitivity of ZnFe_2_O_4_ nanofiber (Figure 5e) sensors to H_2_S, achieving a response of 102 to 1 ppm H_2_S, along with excellent resistance to humidity and a short response time of 12 s. Zhu et al. [119] synthesized porous ZnFe_2_O_4_ nanorods using a microemulsion system with calcination at 500 °C. The resulting ZnFe_2_O_4_ nanorods had a diameter of approximately 50 nm, composed of ZnFe_2_O_4_ nanocrystals (with a diameter of 5–10 nm) arranged linearly. Compared with ZnFe_2_O_4_ nanoparticles, porous ZnFe_2_O_4_ nanorods exhibited superior gas-sensing properties to ethanol at room temperature. The enhanced sensing performance can be ascribed to the random arrangement of the porous nanorods and the existence of interconnected porous channels. These factors significantly augmented the specific surface area of the nanorods, facilitating effective diffusion of the target gas for detection. Additionally, the smaller grain size of ZnFe_2_O_4_ offered a greater number of active sites, matching the thickness of the electron-depleted region, thereby amplifying the response. Li et al. [122] conducted a study where ZnFe_2_O_4_ nanorods (Figure 5f) with a porous structure were synthesized using the hydrothermal method, with ZnFe_2_(C_2_O_4_)_3_ serving as the template. These nanorods were composed of small nanoparticles and exhibited a significant number of surface pores. The porous ZnFe_2_O_4_ nanorods sensor demonstrated a rapid response to acetone, with a response of 52.8 and response/recovery times of 1/11 s at 260 °C for 100 ppm acetone. The exceptional response observed in the porous ZnFe_2_O_4_ nanorods sensor can be attributed to several factors, including the fine nanoparticle size, suitable pore size, and reticular pore structure. These characteristics contribute to enhanced gas adsorption and diffusion, allowing for a rapid response to acetone. However, it is important to note that when the concentration of acetone exceeded 100 ppm, the desorption capacity of the sensing material became insufficient compared with its adsorption capacity. As a result, the sensor exhibited a stable response instead of a further increase in signal intensity.

### 3.3. Nanosheets

The preparation methods for spinel ferrite nanosheets primarily include template hydrothermal [129], sol–gel [127], and spray pyrolysis techniques [128]. The template hydrothermal method can prepare nanosheets with specific pore structures and morphologies, but the demolding step may limit the sample’s morphology and structure [129]. The sol–gel method can prepare spinel ferrite nanosheets with specific compositions and structures, but it tends to introduce impurities [127]. Spray pyrolysis can produce thinner nanosheet films with good lattice matching and crystallinity, but the equipment cost is high and the operation is relatively complex [128].

Nanosheets are a type of two-dimensional nanomaterial characterized by their flat, sheet-like structure. Due to their unique morphology, nanosheets possess a large surface area-to-volume ratio, providing an abundance of reaction sites and diffusion paths for gases to interact with. This increased surface area and availability of reaction sites contribute to improved gas-sensing properties, such as enhanced sensitivity and selectivity. The highly exposed surface of nanosheets allows for efficient gas adsorption and interaction, making them promising candidates for gas-sensing applications.

Singh et al. [126] prepared high-porous CuFe_2_O_4_ cascade nanostructures by sol–gel method. It has a porous structure CuFe_2_O_4_ with pore size between 10–15 nm. The results of the sensing experiments demonstrate that the porous CuFe_2_O_4_ layered structure exhibits a high sensing response of 96% when exposed to LPG at a temperature of 25 °C. Moreover, it demonstrates excellent repeatability and rapid response recovery characteristics. Gao et al. [129] successfully synthesized porous ZnFe_2_O_4_ nanosheets (Figure 5g) by utilizing graphene sheets as a rigid template. The resulting ZnFe_2_O_4_ nanosheets had pores with a size range of 5–50 nm and were composed of nanoparticles with a diameter of approximately 10–20 nm. In comparison to Fe_2_O_3_ nanoparticles, ZnO nanoparticles, and ZnFe_2_O_4_ nanoparticles, the sensor based on ZnFe_2_O_4_ nanosheets exhibited faster response and recovery times (39 s/43 s), higher response (R_a_/R_g_ = 123) and excellent selectivity. The sensor also demonstrated good repeatability and stability. Moreover, the unique mesoporous ZnFe_2_O_4_ nanosheets enabled the detection of H_2_S gases as low as 500 ppb at 85 °C (Figure 5h). The enhanced performance of the ZnFe_2_O_4_ nanosheets can be ascribed to their high specific surface area and porous characteristics. The increased specific surface area provides more active sites for gas molecule adsorption and reaction, enhancing the gas-sensing response. The porous structure of the nanosheets allows for the diffusion of target gas molecules, facilitating their interaction with the sensing material. Additionally, the two-dimensional structure of the nanosheets prevents the aggregation of nanoparticles, ensuring a larger effective surface area for gas sensing and maintaining the structural integrity of the material. Overall, the combination of high specific surface area, porous features, and two-dimensional structure contributes to the enhanced gas-sensing performance of ZnFe_2_O_4_.

### 3.4. Nanospheres

Spinel ferrite nanospheres can be classified into solid spheres [147], hollow spheres [149], core−shell spheres [139], and double-shell (or triple-shell) spheres [153]. They are mainly prepared using solvent thermal methods or metal–organic framework (MOF) [142] methods. In recent years, the template-free solvent thermal method has become the mainstream approach for synthesizing three-dimensional spinel ferrite materials.

Nanospheres typically consist of solid spheres or hollow spheres that can evolve from the core–shell structure. They are characterized by their low density, high specific surface area, pronounced surface activity, and notable stability [148]. Previous research suggests that to achieve a larger specific surface area, it is essential to decrease the size of the nanoparticles. Assembling nanoparticles into nanospheres allows for better control over the size, resulting in larger specific surface areas and higher sensitivity. The enhanced reactivity and gas-sensing performance of nanospheres can be attributed to their increased surface area-to-volume ratio.

**Figure 6:**
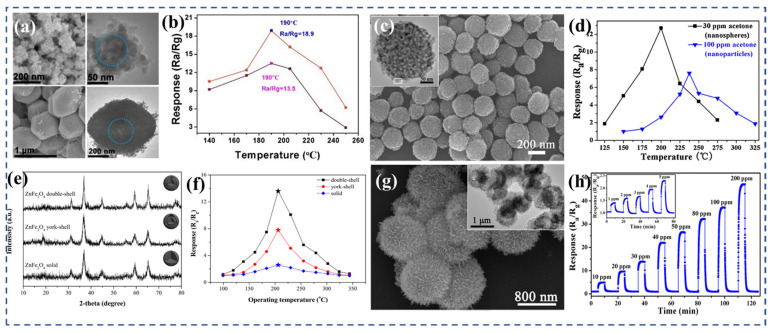
(**a**) SEM and TEM images of the NiFe_2_O_4_ polyhedron. (**b**) The response comparison of sensors to 50 ppm TEA at various temperatures. (**c**) SEM image and TEM image (inset) of the ZnFe_2_O_4_ sphere. (**d**) Comparative analysis of the 30 ppm acetone response of porous ZnFe_2_O_4_ nanospheres and the 100 ppm acetone response of ZnFe_2_O_4_ nanoparticles at varying operating temperatures. (**e**) XRD patterns of ZnFe_2_O_4_ double-shell, yolk–shell, and solid microspheres. (**f**) The sensitivity–temperature characteristics of the ZnFe_2_O_4_ double-shell, yolk–shell, and solid microsphere-based sensors in detecting 20 ppm acetone. (**g**) SEM image and TEM image (inset) of the hierarchical ZnFe_2_O_4_ microspheres. (**h**) Dynamic curve of the gas sensor to acetone with different concentrations at 215 °C. (**a**,**b**) Reproduced with permission [142], copyright 2020, Royal Society of Chemistry. (**c**,**d**) Reproduced with permission [147], copyright 2015, Elsevier B.V. (**e**,**f**) Reproduced with permission [153], copyright 2018, Elsevier B.V. (**g**,**h**) Reproduced with permission [149], copyright 2015, American Chemical Society.

Zhai et al. [142] conducted a study where they synthesized NiFe_2_O_4_ polyhedron structures (Figure 6a) derived from metal–organic frameworks (MOF) using solvothermal synthesis. By altering the solvent composition, they were able to synthesize large NiFe_2_O_4_ polyhedra with a more stable morphology and structure. These large polyhedra exhibited excellent gas-sensing properties for TEA. Notably, they demonstrated a fast response time of 6 s to 50 ppm TEA, an enhanced response value of 18.9 to 50 ppm TEA (Figure 6b), and showed good selectivity and repeatability at relatively low operating temperatures of 190 °C. The fast response rate of the sample can be attributed to its unique dense hollow structure. The hollow structure enables the REDOX reaction between TEA molecules and the material to occur predominantly at the surface/interface, while the interior of the material remains inactive. This reduces the electron conduction path, leading to the observed fast response time. Qu et al. [153] conducted research on the synthesis of ZnFe_2_O_4_ double-shell microspheres using a hydrothermal method and thermal treatment. Figure 6e is the XRD pattern of the yolk–shell, double-shell hollow spheres, and solid microspheres. Compared with the yolk–shell and solid microspheres, the ZnFe_2_O_4_ double-shell hollow spheres not only reduced the operating temperature of the sensor, but also enhanced its acetone sensitivity because of the improved crystallinity and larger specific surface area. The sensor displayed a response of 2.6 to 5 ppm acetone at 206 °C (Figure 6f), with a response time of 6 s and a recovery time of 10 s. Furthermore, it is noteworthy that the detection limit for acetone achieved by the sensor was reported to be 0.13 ppm. This value is significantly below the established risk level for life and health, which is 20,000 ppm. Additionally, it is well below the diagnostic threshold for diabetes, which is set at 0.8 ppm. This indicates the high sensitivity and potential of the sensor in accurately detecting and monitoring acetone levels in various applications.

Zhou et al. [147] successfully synthesized porous ZnFe_2_O_4_ nanospheres (Figure 6c) using a template-free solvothermal method, followed by annealing at 400 °C. These nanospheres consisted of numerous nanoparticles and possessed a pore size ranging from 10 to 20 nm. The distinctive porous spherical structure greatly improved the sensor’s acetone sensing performance. The response value for 30 ppm acetone reached 11.8, which is 2.5 times higher compared with that for the ZnFe_2_O_4_ nanoparticles (Figure 6d). A swift response time of 9 s showcased its ability to promptly detect and react to variations in the target gas. However, the recovery time was relatively longer, taking 272 s. Subsequently, zhou et al. [149] employed a template-free solvent-heat treatment followed by heat treatment at 400 °C for 2 h to fabricate ZnFe_2_O_4_ hollow microspheres assembled with nanosheets (Figure 6g). The nanosheets within the microspheres had an average thickness of 20 nm, while the hollow microspheres themselves had diameters ranging from 0.9 to 1.1 μm. The hollow flower-like structure offered multitudes of adsorption/reaction sites, and the presence of diffusion channels, primarily distributed in the aperture range of 2 to 50 nm, facilitated the diffusion of target gases. At an operating temperature of 215 °C, the sensor exhibited a response value of 37.3 to 100 ppm acetone (Figure 6h) and demonstrated good long-term stability. However, under the same conditions, the response to ethanol was also high, measuring at 27.0. The presence of layered hollow structures in semiconductor oxides can enhance the diffusion of target gases, making them advantageous for gas-sensor applications.

## 4. Doping

Element doping is indeed a powerful strategy to enhance the structure and performance of spinel ferrite materials, and there has been a growing interest in this research area recently. While earlier studies on spinel ferrite doping mostly concentrated on applications such as electrodes and magnetism, recent advancements have shed light on the importance of doping for optimizing gas-sensing properties. However, not all metallic elements are suitable for doping in spinel ferrite materials. Preferably, elements with donor characteristics (high valence elements that can donate electrons) or acceptor characteristics (low valence elements that can accept electrons) are used for modification. Doping in spinel ferrite materials can occur in two forms. The first form of doping involves displacement, where the M^2+^ (A site) and Fe^3+^ (B site) ions in the spinel ferrite are replaced by the doping elements. This changes the composition of the spinel ferrite and can affect its properties, such as A-site doping [155], B-site doping [156], and AB-site doping [157]. The second involves the incorporation of doping elements into the tetrahedral and octahedral interstices of MFe_2_O_4_ crystals. This results in a solid solution structure, where the doping elements are homogeneously dispersed within the host material [158]. Doping can significantly alter the composition and microstructure of spinel ferrite materials, influencing characteristics such as crystallinity [159]. These changes can, in turn, affect the reference resistance [160] and gas-sensing performance [161] of the ferrite-based gas sensors. For instance, doping can enhance the sensitivity [162], selectivity [163], response speed [28], and stability [164] of the sensors. In this section, we will review the latest research progress on element doping in spinel ferrite materials and its influence on their gas-sensing properties (Table 5, Table 6, Table 7, Table 8 and Table 9). The focus will be on how different doping elements can affect the sensor’s performance, the optimal doping concentrations, and the underlying mechanisms behind these effects. This review will provide valuable insights for the design and fabrication of high-performance ferrite-based gas sensors.

### 4.1. A Site Doping

**Figure 7:**
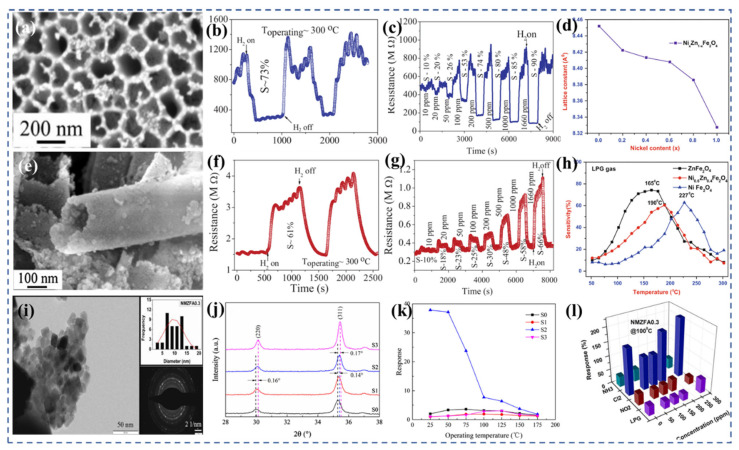
(**a**) FESEM image of embedded Mg_0_._5_Zn_0_._5_Fe_2_O_4_ nanotubes. (**b**) Resistance transients of embedded Mg_0_._5_Zn_0_._5_Fe_2_O_4_ nanotubes towards H_2_ (∼1660 ppm). (**c**) Dynamic curve of the resistance embedded Mg0._5_Zn_0_._5_Fe_2_O_4_ nanotube sensors to the 10–1660 ppm range of H_2_ at ∼350 °C. (**d**) Variations of the lattice constant with Ni content of NiZnFe_2_O_4_ system. (**e**) SEM image of the isolated Mg_0_._5_Zn_0_._5_Fe_2_O_4_ nanotubes. (**f**) Resistance transient of isolated Mg_0_._5_Zn_0_._5_Fe_2_O_4_ nanotubes to 1660 ppm H_2_. (**g**) Dynamic curve of the resistance isolated Mg0._5_Zn_0_._5_Fe_2_O_4_ nanotube sensors to the 10–1660 ppm range of H_2_ at 350 °C. (**h**) Response of sensors based on Ni_x_Zn_1−x_Fe_2_O_4_ (x = 0, 0.6, 1.0) to LPG gas at different operating temperatures. (**i**) The TEM images and SAED pattern of Ni_0_._7−x_Mn_x_Zn_0_._3_Fe_2_O_4_. (**j**) Small-range XRD patterns of the pure ZFNPs and Cu-ZFNPs with different Cu concentrations. (**k**) The variation in sensitivity with operating temperatures of pure ZFNPs and Cu-ZFNPs for 5 ppm H_2_S. (**l**) The response−concentration plots of Ni_0_._4_Mn_0_._3_Zn_0_._3_Fe_2_O_4_ towards different test gases. (**a**–**c**,**e**–**g**) Reproduced with permission [155], copyright 2013, Elsevier B.V. (**d**,**h**) Reproduced with permission [90], copyright 2015, Springer Nature. (**i**,**l**) Reproduced with permission [199], copyright 2022, Elsevier B.V. (**j**,**k**) Reproduced with permission [187], copyright 2019, Elsevier B.V.

Compounds of the MFe_2_O_4_ type, where M represents elements such as Mg, Cu, Zn, Ni, and Co, are widely utilized in the field of sensors due to their favorable surface activity. The study conducted by Mukherjee et al. [155] presents an interesting perspective on how the morphology and structure of ferrite-based materials can influence their gas-sensing properties. In their research, they synthesized one-dimensional Mg_0_._5_Zn_0_._5_Fe_2_O_4_ hollow tubes using a wet chemical process assisted by an alumina template. They evaluated the gas-sensitive properties of two versions of these nanotubes: one version was embedded in a porous alumina template (Figure 7a) and the other was isolated and coated on a quartz substrate (Figure 7e). The nanotubes exhibited good responsiveness to H_2_, CO, and N_2_O gases in both configurations. Interestingly, they observed a difference in the behavior of the nanotubes based on their configuration. Regardless of the type of test gas, the concentration of the test gas, or the operating temperature, the embedded nanotubes consistently behaved as N-type semiconductors. N-type semiconductors are characterized by an excess of electrons (Figure 7b,c). On the other hand, the isolated nanotubes behaved as P-type semiconductors (Figure 7f,g), which are characterized by a deficiency of electrons or an excess of “holes” for the electrons. This inversion from N-type to P-type dominance of carriers, when going from embedded to isolated nanotubes, is a significant finding. It suggests that the electronic properties of ferrites can be customized by changing their surface-to-volume ratio. In other words, by altering the physical configuration of the ferrites (from embedded to isolated), it is possible to control their semiconductor behavior. This finding opens up new possibilities for the design and fabrication of ferrite-based gas sensors, as it introduces an additional degree of tunability in their properties.

Dalawai et al. [90] prepared Ni_x_Zn_1−x_Fe_2_O_4_ (x = 0, 0.2, 0.4, 0.6, 0.8, and 1.0) using the oxalic acid co-precipitation method. With the increase in nickel content in Ni-Zn ferrite, the bond length (A-O) and ionic radius (r_A_) at site A decreased (Figure 7d), while the bond length (B-O) and ionic radius (r_B_) at site B remained unchanged. Infrared spectroscopy revealed two major absorption bands near 400 and 600 cm^−1^, corresponding to tetrahedral and octahedral locations, respectively. Compared with LPG and Cl_2_, ZnFe_2_O_4_ thick films showed a higher sensitivity to ethanol (82%) (Figure 7h), better response time (30 s), and better recovery time (90 s). NiFe_2_O_4_ thick film has a good sensitivity (63%), good response (30 s) and good recovery time (70 s) to LPG. Compared with LPG, Ni_0_._6_Zn_0_._4_Fe_2_O_4_ displayed a higher sensitivity towards Cl_2_ and ethanol gases. Zhang et al. [187] conducted a study where they synthesized Cu-doped ZnFe_2_O_4_ nanoparticles (Cu-ZFNPs) using a hydrothermal method. Interestingly, the addition of copper did not significantly alter the size of the nanoparticles, which remained around 50 nm for both the pure and Cu-doped ZFNPs. Figure 7j shows the XRD patterns of the pure ZFNPs and Cu-ZFNPs with different Cu concentrations However, the gas-sensing performance of the nanoparticles was notably affected by copper doping. The Cu-ZFNPs exhibited a superior performance in detecting H_2_S gas compared with the pure ZFNPs, particularly at lower temperatures. This proves that the introduction of copper into the ZnFe_2_O_4_ nanoparticles improved their sensitivity to H_2_S gas, highlighting the effectiveness of element doping in optimizing the properties of spinel ferrite materials. The best gas-sensing performance was achieved with Cu-ZFNPs containing an appropriate concentration of copper. These nanoparticles demonstrated a maximum response of 37.9 to 5 ppm H_2_S at room temperature (Figure 7k). The sensor also exhibited rapid response and recovery times, taking only 10 s to respond to the presence of H_2_S and 210 s to recover after the gas was removed.

Using the co-precipitation method, Mondal et al. [201] conducted a study where they synthesized Cu_0_._5_Ni_0_._25_Zn_0_._25_Fe_2_O_4_ nanoparticles and Cu_0_._25_Ni_0_._5_Zn_0_._25_Fe_2_O_4_ nanoparticles. At ambient room temperature, both sensors demonstrated exceptional responsiveness to acetone and ethanol. The inclusion of Cu in Cu_0_._5_Ni_0_._25_Zn_0_._25_Fe_2_O_4_ resulted in a noteworthy enhancement in sensitivity to acetone, reaching an impressive 77%, while the introduction of Ni in Cu_0_._25_Ni_0_._5_Zn_0_._25_Fe_2_O_4_ improved the sensitivity to ethanol to 75%. These findings suggest that the addition of specific transition metal elements, such as copper and nickel, enhances the gas-sensing properties of the ferrite nanoparticles, making them promising materials for the detection of acetone and ethanol gases. Gauns et al. [199] fabricated a thick film of Ni_0_._4_Mn_0_._3_Zn_0_._3_Fe_2_O_4_ (Figure 7i) on a glass substrate for the detection of Cl_2_. The thick ferrite film composed of x = 0.3 showed a high selective response to Cl_2_ gas at 100 °C. For 300 ppm of Cl_2_ gas, the response was 212% (Figure 7l). The reaction time was less than 10 s and the recovery time was less than 15 s.

**Table 6:** Summary of the reported spinel ferrite B-site doping-based gas sensors.

Materials	Synthesis	Morphology	Gas	O.T. (°C)	Conc. (ppm)	Response	t_res_/t_rec_	LOD	Refs.
MgFe_1_._98_Mo_0_._02_O_4_	Auto-combustion	Nanoparticles (310 nm)	Acetone	380	500	0.65 c	180 s/	-	[165]
Li_0_._5_Fe_2_._45_Sm_0_._05_O_4_	Sol–gel self-combustion	Nanoparticles (200 nm)	Methanol	340	200	0.86 c	-	-	[202]
CuCe_0_._04_Fe_1_._96_O4	Molten-salt	Nanoparticles (10 nm)	LPG	275	2000	0.86 c	5 s/68 s	-	[203]
CoFe_1_._96_Ce_0_._04_O4	Molten-salt	Nanoparticles (20 nm)	Acetone	225	100	1.77 b	45 s/70 s	-	[204]
NiLaFe_2_O_4_	Co-precipitation	Nanoparticles (9.26 nm)	NH_3_	35	50	786 a	163/64 s	-	[205]
Bi-Co ferrite	Sol–gel	Nanoparticles (6.5–89 nm)	NO_2_	230	200	0.34 c	31/29 s	-	[186]
MgFe_1_._88_Ce_0_._12_O_4_	Glycine combustion	Thick film	Acetone	325	1000	0.94 c	-	-	[156]
CoSm_0_._1_Fe_1_._9_O_4_	Solvothermal	Nanoparticles	LPG	225	10,000	846 c	-	-	[206]
MgCe_0_._2_Fe_1_._8_O_4_	Glycol-thermal	Nanoparticles	Acetone	225	100	500 a	-	-	[207]
1.5% Sn-BiFe_2_O_4_	Sol–gel	Nanoparticles	HCHO	280	1	3.05 b	2.7 s/25 s	100 ppb	[208]
1 wt.% La-CoFe_2_O_4_	Spray-deposited	Thin films	NH_3_	RT	200	0.99 c	44/53 s	-	[209]

a Response is defined as R_a_/R_g_; b Response is defined as R_g_/R_a_; c Response is defined as ∆R/R_a_.

### 4.2. B Site Doping

**Figure 8:**
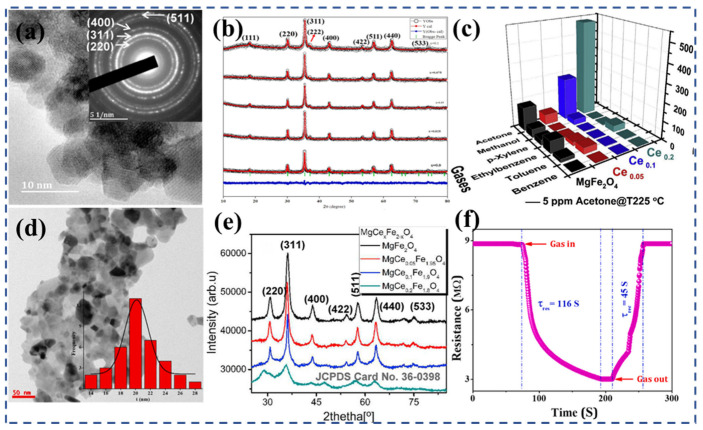
(**a**) HRTEM images of MgCe_0_._2_Fe_1_._8_O_4_. (**b**) The XRD patterns of MgCe_x_Fe_2−x_O_4_. (**c**) Responses of MgCe_x_Fe_2−x_O_4_ nanoferrites (x = 0, 0.05, 0.1, and 0.2) to various gas with 100 ppm. (**d**) TEM image of CZLF ferrite with x = 0.1. (**e**) XRD pattern of La^3+^-CZLF powders. (**f**) The resistance plot of a sensor based on Co_0_._7_Zn_0_._3_La_0_._1_Fe_1_._9_O_4_. (**a**–**c**) Reproduced with permission [207], copyright 2020, Elsevier B.V. (**d**–**f**) Reproduced with permission [214], copyright 2022, Elsevier B.V.

Spinel ferrite, represented by the formula (M^2+^)(Fe^3+^)_2_O_4_, adopts a face-centered cubic crystal structure. It can be classified into three types: normal spinel, inverse spinel, and mixed spinel. The arrangement of divalent and trivalent metal ions in tetrahedral and octahedral sites within the crystal lattice determines the spinel classification [210]. The introduction of rare earth ions (RE) as substitutions for a small portion of iron can have significant effects on the electrical and magnetic properties of spinel ferrite. For example, the introduction of Ce, which involves the coupling of 3d–4f interactions, leads to changes in the electrical and magnetic behaviors. Furthermore, Ce substitution can also impact the distribution of cations within the spinel lattice, resulting in alterations to its structural, magnetic, physicochemical, and electrical properties [211]. Other rare earth elements, when substituted into the spinel structure, can similarly induce changes in structural, magnetic, and electrical properties, although the specific effects may differ from those observed with Ce^3+^ [212]. Mkwae et al. [207] conducted a study where they prepared MgCe_x_Fe_2−x_O_4_ (0 < x < 0.2) nanoparticles (Figure 8a). X-ray diffraction (Figure 8b) analysis confirmed that the sample containing a lower concentration of Ce formed a pure cubic spinel phase. However, with higher Ce doping (x > 0.2), the formation of a secondary phase was observed. The grain size of the compounds ranged from 2.2 nm to 15.3 nm. As the Ce concentration increased, the spin state of ^57^Fe Mossbauer transitioned from an ordered state to a paramagnetic state. The MgCe_x_Fe_2−x_O_4_ nano-ferrite exhibited a high sensitivity and selectivity towards the 100 ppm acetone vapors, with a response concentration exceeding 500 at 225 °C (Figure 8c). The sensor also demonstrated excellent repeatability, reversibility, and stability over a period of 120 days.

**Table 7:** Summary of the reported spinel ferrite AB site doping-based gas sensors.

Materials	Synthesis	Morphology	Gas	O.T. (°C)	Conc. (ppm)	Response	t_res_/t_rec_	LOD	Refs.
Mg_0_._9_Sn_0_._1_Mo_0_._02_ Fe_1_._98_O4	Auto-combustion	Nanoparticles	ethanol	380	500	0.64 c	-	-	[165]
N_i0_._99_Co_0_._01_Mn_0_._02_Fe_1_._98_O4	Self-combustion	Nanoparticles	acetone	215	500	4.5 c	-	-	[213]
Co_0_._7_Zn_0_._3_Fe_1_._975_ Gd_0_._025_O4	Sol–gel	Nanoparticles	H_2_S	RT	50	0.4 d	11 s/5 s	-	[157]
Co_0_._7_Zn_0_._3_La_0_._1_ Fe_1_._9_O_4_	Sol–gel	Nanoparticles (20 nm)	NH_3_	RT	200	0.87 c	116 s/45 s	-	[214]
Zn_0_._7_Mn_0_._3_Gd_0_._025_Fe_1_._975_O_4_	Co-precipitation	Nanoparticles (20–30 nm)	acetone	RT	saturated	0.53 c	36 s/56 s	-	[215]

c Response is defined as ∆R/R_a_; d Response is defined as ∆R/R_g_.

**Table 8:** Summary of the reported noble metal-decorated spinel-ferrite-based gas sensors.

Materials	Synthesis	Morphology	Gas	O.T. (°C)	Conc. (ppm)	Response	t_res_/t_rec_	LOD	Refs.
Au/NiFe_2_O_4_	Solid-state reaction	Nanoparticles	H_2_S	300	5	35.8 b	-	-	[216]
Au/NiFe_2_O_4_	Co-precipitation	Nanoparticles	C_6_H_5_CH_3_	350	1000	15.8 b	-	-	[217]
Au/ZnFe_2_O_4_	Solvothermal	Yolk–shell Microspheres	H_2_S	RT	200	65.9 a	46/629 s	-	[218]
ZnO/ZnFe_2_O_4_/Au	Electrospinning, atomic layer deposition and solution reaction	Hollow meshes	Acetone	225	100	30.3 a	1/59 s	-	[219]
Au/ZnFe_2_O_4_	Solution-phase deposition	Yolk–shell Spheres	C_6_H_5_Cl	150	10	90.9 a	-	100 ppb	[220]
Au/ZnFe_2_O_4_	Hydrothermal	Nanoparticles	Acetone	120	40	26 a	4/69 s	-	[221]
ZnO/ZnFe_2_O_4_/Au	Hydrothermal and Co-precipitation	Yolk–shell microspheres assembled from nanosheets	Acetone	206	100	18.18 a	4/23 s	0.7 ppm	[222]
Ag/NiFe_2_O_4_	Solid-state reaction	Nanoparticles	Acetone	-	1000	43 a	1/10 s	-	[223]
Ag/ZnFe_2_O_4_	Hydrothermal	Hollow sphere	Acetone	175	100	33.8 a	17/148 s	-	[224]
Pd/Co_0_._8_Ni_0_._2_Fe_2_O_4_	Sol–gel	Nanoparticles	NH_3_	210	200	0.91 c	20 s/-	-	[225]
Pd/MgFe_2_O_4_	Molten salt	Nanoparticles (15–20 nm)	LPG	200	200	432 a	-	-	[226]
Pd/NiFe_2_O_4_	Spray pyrolysis	Nanoparticles	Ethanol	325	15	4.15 c	3/13 s	-	[158]
Pd/NiFe_2_O_4_	Spray pyrolysis	Thin films	Cl_2_	375	5	6.9 d	-	-	[227]
Pd/Co_0_._55_Zn_0_._45_Fe_2_O_4_	Hydrothermal	Nanoparticles	H_2_	275	5000	0.99 c	25/3 s		[228]
Pt/CuFe_2_O_4_	Electrospinning	Nanotubes	Acetone	300	100	16.5 a	-	-	[229]
Ru/NiFe_2_O_4_	Co-precipitation	Nanoparticles (0.48 nm)	H_2_S	100	50	1.39 b	-	-	[230]

a Response is defined as R_a_/R_g_; b Response is defined as R_g_/R_a_; c Response is defined as ∆R/R_a_; d Response is defined as ∆R/R_g_.

### 4.3. AB Site Doping

Rezlescu et al. [165] conducted a study where they prepared Mg_1−x_Sn_x_Mo_y_Fe_2−y_O_4_ (x = 0, 0.1, and y = 0, 0.02) ferrites using metal nitrate as the raw materials using the self-combustion method. The introduction of Sn and Mo ions induced structural changes in terms of grain size and porosity. Specifically, the sample containing tin exhibited the highest porosity, with particle sizes around 100 nm. When Sn ions partially replaced Mg in MgFe_2_O_4_ ferrite, the resistivity of the material improved by approximately two orders of magnitude. The samples were subjected to testing to evaluate their sensing capabilities towards reducing gases, specifically ethanol and acetone. The gas sensitivity was found to depend largely on the type of substituted ion and the specific gas being detected. Overall, all ferrites exhibited a higher sensitivity to acetone compared with ethanol. Among all of the ferrites tested, Mg_0_._9_Sn_0_._1_Fe_2_O_4_ demonstrated the highest sensitivity to acetone. These findings highlight the potential of Mg_0_._9_Sn_0_._1_Fe_2_O_4_ ferrite as a highly sensitive material for the detection of acetone gas. Mugutkar et al. [214] synthesized Co_0_._7_Zn_0_._3_La_x_Fe_2−2x_O_4_ (x = 0–0.1) nanoparticles (Figure 8d) using the sol–gel method. The XRD pattern (Figure 8e) of ferrite powder was refined using the Rietveld technique, and it was found that a single-phase spinel structure was formed. Through the analysis of the gas-sensitive properties, the response of the Co_0_._7_Zn_0_._3_La_x_Fe_2−2x_O_4_ sensor was 0.87 towards 200 ppm NH_3_ at RT, with a short response and recovery time of 116 and 45 s (Figure 8f), respectively.

### 4.4. Noble Metal Doping

Currently, the noble metals widely utilized in gas-sensing applications encompass Pt, Pd, Au, Ag, and Ru, as well as their bimetallic composites. The enhancement of gas-sensing performance can be attributed to two key mechanisms: the electronic sensitization effect achieved by constructin metal−semiconductor contact [231] and the chemical sensitization effect stemming from the spillover phenomenon [232]. These mechanisms work in tandem, facilitating rapid interaction between noble-metal-decorated semiconductor spinel ferrite and target gases, while also effectively lowering the work temperatures by reducing the activation energy required for gas sensing.

**Table 9:** Summary of the reported other element doping spinel-ferrite-based gas sensors.

Materials	Synthesis	Morphology	Gas	O.T. (°C)	Conc. (ppm)	Response	t_res_/t_rec_	LOD	Refs.
V-ZnFe_2_O_4_	Citrate pyrolysis	Nanoparticles	Acetone	300	100	23 a	-	-	[233]
Zr-CaFe_2_O_4_	Solid-state reaction	Nanoparticles	CO_2_	350	5000	3.3 a	-	-	[234]
In-CuFe_2_O_4_	Co-precipitation	Thin film	LPG	25	5 vol%	0.3715 c	229 s/-	-	[184]
Sb-ZnFe_2_O_4_	Spray pyrolysis	Microporous spheres	n-butanol	250	100	33.5 a	4 s/250 s	-	[235]
V-NiFe_2_O_4_	Co-precipitation	Nanoparticles	NO	RT	200	43 a	5 s/-	-	[236]
W-CoFe_2_O_4_	Sol–gel	Nanoparticles	Acetone	350	2000	1.45 c	-		[237]

a Response is defined as R_a_/R_g_; c Response is defined as ∆R/R_a_.

**Figure 9:**
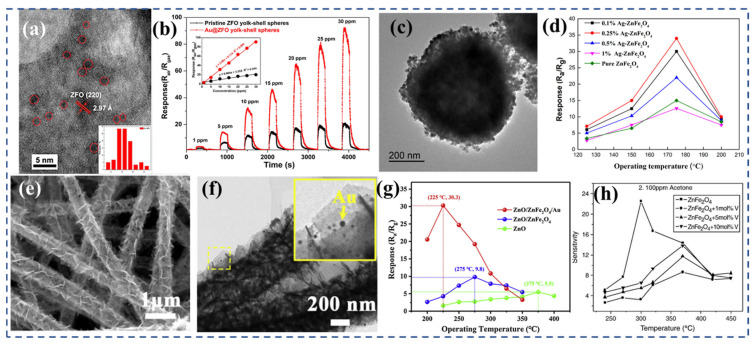
(**a**) HRTEM images of the Au nanoparticles/ZFO yolk–shell spheres and the inset is the size distribution of Au nanoparticles (marked with red circle). (**b**) Dynamic curve of the gas sensor based on the ZFO and Au/ZFO sphere to CB with different concentrations at 150 °C. (**c**) TEM images of 0.25 wt.% Ag/ZnFe_2_O_4_. (**d**) The effect of operating temperatures of the Ag/ZnFe_2_O_4_-sensor on the various gas responses of the sensors to 100 ppm acetone vapor at 125–200 °C. (**e**) SEM and (**f**) TEM images of ZnO/ZnFe_2_O_4_/Au ternary heterostructure. (**g**) Responses–temperature characteristics of the ZnO/ZnFe_2_O_4_/Au sensors to 100 ppm acetone. (**h**) The responses of sensors to ZnFe_2_O_4_ thick films vs. the content of V doping. (**a**,**b**) Reproduced with permission [220], copyright 2019, American Chemical Society. (**c**,**d**) Reproduced with permission [224], copyright 2018, Elsevier B.V. (**e**–**g**) Reproduced with permission [219], copyright 2019, Elsevier B.V. (**h**) Reproduced with permission [233], copyright 2006, Elsevier B.V.

Li et al. [220] conducted a study wherein they utilized the liquid phase deposition precipitation method to prepare a ZnFe_2_O_4_ egg yolk–shell ball structure consisting of ultra-thin nanosheets and ultra-small nanoparticles. The surface of this structure was adorned with nanoscale gold particles, each with a diameter ranging from 1 to 2 nm. The experimental results revealed a significant four-fold increase in response (R_air_/R_gas_ = 90.9) for the Au/ZnFe_2_O_4_ sensor when exposed to 10 ppm chlorobenzene at 150 °C (Figure 9b), compared with the original ZFO sensor. Furthermore, the Au/ZnFe_2_O_4_ sensor demonstrated excellent selectivity and exhibited the potential for application in chlorobenzene monitoring. The introduction of nanoscale gold particles onto the surface of the ZFO yolk–shell balls (Figure 9a) resulted in electronic and chemical sensitization effects, thereby enhancing the chlorobenzene sensing performance of the ZnFe_2_O_4_ yolk–shell balls. Additionally, density functional theory (DFT) calculations were employed to corroborate the findings, confirming that the presence of gold nanoparticles on the surface of ZnFe_2_O_4_ increased electron density, exhibited a higher adsorption energy, and facilitated net charge transfer. These factors collectively contributed to the heightened sensing response of the sensor towards chlorobenzene. Zhang et al. [224] employed a hydrothermal method to introduce Ag into ZnFe_2_O_4_ hollow structures (Figure 9c) composed of stacked nanosheets. The addition of Ag altered the surface structure, but did not significantly affect the size of the hollow structures. At a temperature of 175 °C, the sensor based on 0.25 wt.% Ag-doped ZnFe_2_O_4_ (Ag/ZnFe_2_O_4_) exhibited a superior sensing performance compared with the pure ZnFe_2_O_4_ sensor (Figure 9d). This improvement in performance can be attributed to the suitable hollow structure and the activation effect of Ag. Ag/ZnFe_2_O_4_ sensors show promising potential for detecting low concentrations of acetone in the parts per million range. Additionally, these sensors demonstrate good gas selectivity to acetone and minimal influence from humidity. However, further research and improvement are needed to address the long-term stability of Ag/ZnFe_2_O_4_ sensors.

Li et al. [219] successfully synthesized ZnO/ZnFe_2_O_4_/Au heterostructures (Figure 9e,f) with a porous mesh structure using a three-step method (a combination of electrospinning, atomic layer deposition, and solution reaction). The resulting ZnO/ZnFe_2_O_4_/Au structures exhibited a porous mesh-like morphology. The composite structure comprised of a uniform ZnO nanotube skeleton measuring 50 nm, ultra-thin ZnFe_2_O_4_ nanosheets with a thickness of 10 nm, and well-dispersed Au nanoparticles. It had the characteristics of a large specific surface area, porous structure, ultra-thin thickness and high catalytic activity. The gas-sensing results show that the sensor based on the ZnO/ZnFe_2_O_4_/Au nanonet had the highest sensing response (30.3), a significantly enhanced selectivity, and a faster response/recovery speed (1 s/59 s). The response of ZnO/ZnFe_2_O_4_/Au to acetone was about three times higher than that of ZnO/ZnFe_2_O_4_ composites and 5.5 times higher than that of the original ZnO (Figure 9g). The enhanced sensing performance was mainly due to the increase in the surface active sites of AuNPs, the obvious resistance modulation effect, and the excellent sensitization ability.

### 4.5. Other Element Doping

Doping refers to the process of introducing impurity atoms into a material, which can have various effects on the lattice and structure of the host material. One effect of doping is the alteration of the lattice constant, which is the spacing between the atoms in the crystal lattice. The presence of dopant atoms can disrupt the regular arrangement of atoms in the lattice, leading to changes in the lattice constant. Furthermore, doping can also introduce structural defects into the matrix material. These defects can include vacancies, where atoms are missing from lattice sites, or interstitials, where dopant atoms occupy spaces between lattice sites [238]. These defects can affect the overall structure and properties of the material, such as its electrical conductivity or optical properties. In addition to changing the lattice constant and introducing structural defects, doping can also regulate the charge exchange behavior of the material [239]. Doped ions often have multiple valence states, meaning they can exist in different charge states depending on the electron configuration [184]. When doped ions occupy equivalent lattice locations, they can undergo charge exchange with neighboring ions, leading to changes in the electronic properties of the material. This charge exchange behavior can influence the material’s conductivity, magnetism, or other electronic properties [236,237]. Overall, doping is a versatile technique that can be used to modify the lattice, introduce defects, and regulate the charge exchange behavior in materials, thereby tailoring their properties for specific applications. Jiang et al. [233] conducted a study where they prepared ZnFe_2_O_4_ nanoparticles and vanadium (V)-doped ZnFe_2_O_4_ nanoparticles using citrate pyrolysis. Interestingly, the particle size of the spherical particles remained unaffected by the V content added. However, as the V content increased, the resistance of the thick film based on ZnFe_2_O_4_ decreased. The study also revealed that the addition of V had varying effects on the sensitivity to different VOCs (Figure 9h). The sensitivity to ethanol and acetone was significantly reduced due to the addition of V. However, at higher temperatures, the addition of V notably improved the sensitivity to benzene, toluene, and xylene. These findings suggest that V doping in ZnFe_2_O_4_ nanoparticles can have a selective impact on the sensitivity to different VOCs. While the sensitivity to ethanol and acetone decreased, the sensitivity to benzene, toluene, and xylene improved, particularly at elevated temperatures.

## 5. Heterostructure

In Section 3 and Section 4, it has been discussed how the gas-sensitive performance of spinel ferrite sensors can be enhanced through the manipulation of their morphology or the introduction of doping elements. However, to achieve the desired properties, researchers have explored the development of spinel ferrite composites, which find more extensive applications in the fields of photocatalysis and sensing. Consequently, the objective of this section is to provide a review of the latest research on spinel ferrite composites and to present the impact of these two types of composites on the gas-sensitive properties (Table 10, Table 11 and Table 12). The development of spinel ferrite composites has gained significant attention due to their potential to synergistically enhance the gas-sensitive performance. These composites often involve combining spinel ferrite with other materials such as metal oxides, carbon-based materials, or polymers. The unique properties of these composite materials can be leveraged to improve the gas-sensing properties of spinel ferrite sensors. For example, metal-oxide-based spinel ferrite composites have demonstrated an improved gas-sensing performance due to the enhanced specific surface area and increased active sites provided by the metal oxide component. The combination of spinel ferrite with carbon-based materials, such as graphene or carbon nanotubes, can enhance the electrical conductivity and provide additional adsorption sites, leading to enhanced gas-sensing capabilities. In summary, the development of spinel ferrite composites has opened up new avenues for enhancing the gas-sensitive properties of spinel ferrite sensors. These composites, whether metal-oxide-based, carbon-based, or incorporating polymers, offer unique advantages that can be leveraged to achieve an improved gas-sensing performance.

### 5.1. Other MOSs/Ferrite

There are primarily two methods for synthesizing heterostructures between other metal oxides and spinel ferrite: the one-step method [283] and the multi-step method [258]. The one-step method can yield highly uniform heterostructures, forming microscopic heterojunctions, but it is challenging to control the ratio of the two phases [271]. The multi-step method allows for more precise control in different synthesis stages, including the reaction conditions, proportions, and reaction time, to obtain the desired product properties and structures. However, it increases the duration and cost of the synthesis process [273].

**Figure 10:**
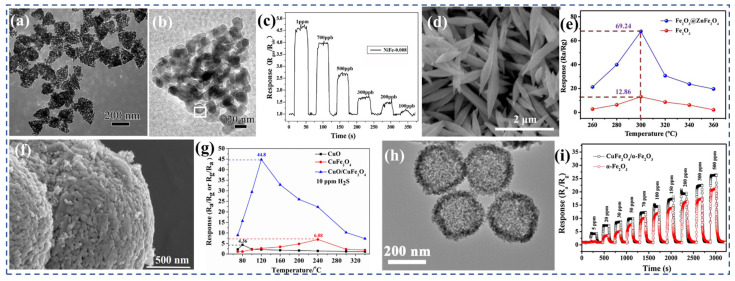
(**a**) SEM and (**b**) TEM images of NiO/NiFe_2_O_4_. (**c**) Dynamic curve of the NiO/NiFe_2_O_4_-sensor to formaldehyde with different concentrations at 240 °C. (**d**) SEM image of Fe_2_O_3_/ZnFe_2_O_4_. (**e**) Comparative analysis of the 100 ppm TEA response among sensors based on Fe_2_O_3_ spindles and Fe_2_O_3_/ZnFe_2_O_4_ at varying operating temperatures. (**f**) SEM images of CuO/CuFe_2_O_4_. (**g**) Comparative analysis of the 100 ppm TEA response among sensors based on CuO microspheres, CuFe_2_O_4_ nanoparticles, and CuO/CuFe_2_O_4_ heterostructure at varying operating temperatures. (**h**) TEM images of Fe_2_O_3_/CuFe_2_O_4_ composite. (**i**) Gas-sensing performances of hollow Fe_2_O_3_ and CuFe_2_O_4_/Fe_2_O_3_-2-composite-based sensors under various concentrations of acetone ranging from 5 to 500 ppm. (**a**–**c**) Reproduced with permission [258], copyright 2020, Elsevier B.V. (**d**,**e**) Reproduced with permission [254], copyright 2020, Elsevier B.V. (**f**,**g**) Reproduced with permission [248], copyright 2018, Elsevier B.V. (**h**,**i**) Reproduced with permission [29], copyright 2018, Elsevier B.V.

Xu et al. [258] conducted a study in which they prepared NiO/NiFe_2_O_4_ nanocomposites using a straightforward two-step hydrothermal method. The nanocomposites consisted of NiO nano-tetrahedrons with numerous NiFe_2_O_4_ nanoparticles dispersed on their outer surface (Figure 10a,b), forming p–p type heterojunctions. By adjusting the amount of Fe added during the synthesis process, the Fe to Ni ratio was optimized. The nanocomposite designated as NiFe-0.008 exhibited a remarkable gas-sensing performance (Figure 10c), with a high response of 19.1 towards 50 ppm formaldehyde smoke at 240 °C. Additionally, it displayed a low detection limit of 200 ppb and demonstrated good long-term stability. Comparatively, the optimized NiFe-0.008 nanocomposite outperformed individual NiO nano-tetrahedrons (with a response of 11.6 at 250 °C) and NiFe_2_O_4_ nanoparticles (with a response of 6.8 at 300 °C) in terms of the gas-sensing performance. These findings highlight the improved response performance achieved by the optimized NiFe-0.008 nanocomposite. Hu et al. [248] conducted a study where they modified CuO microspheres by incorporating CuFe_2_O_4_ nanoparticles (Figure 10f), resulting in CuFe_2_O_4_/CuO heterostructures. These heterostructures exhibited a high sensitivity to hydrogen H_2_S. The researchers investigated the relationship between the mass ratio of CuFe_2_O_4_ to CuO and the operating temperature to optimize the sensor’s response to H_2_S.The results of the study demonstrate that the optimized CuFe_2_O_4_/CuO heterostructures exhibited a significantly enhanced response to 10 ppm H_2_S at 240 °C (Figure 10g), reaching approximately 20 times that of the initial CuO microspheres. Moreover, the optimized heterostructures showed excellent fast response and recovery abilities. These findings suggest that the incorporation of CuFe_2_O_4_ nanoparticles into CuO microspheres can effectively enhance the gas-sensing performance of the sensor towards H_2_S. The optimized CuFe_2_O_4_/CuO heterostructures demonstrated a substantial improvement in sensitivity compared with the preliminary CuO microspheres, making them promising candidates for the detection of H_2_S gas. Balaji et al. [263] conducted a study in which they synthesized SnO_2_ composite Mn_1−x_CuFe_2_O_4_ (x = 0, 0.5, and 1.0) nanocomposites with an equal mass percentage using the chemical coprecipitation method. The addition of SnO_2_ to copper-substituted manganese ferrite resulted in an increase in grain size and a decrease in strain value. The morphological analysis revealed that the average particle size of the ferritic materials decreased linearly with the decrease in Mn^2+^ concentration. The presence of SnO_2_ on the surface of Cu-Mn ferrite led to an increase in particle size and a weakening of the magnetic properties. Furthermore, the addition of SnO_2_ to MnFe_2_O_4_ and Mn_1−x_Cu_x_Fe_2_O_4_ enhanced the sensitivity of the gas sensor. MnFe_2_O_4_ exhibited resistance to oxygen and carbon dioxide, while SnO_2_-CuFe_2_O_4_ showed a weak sensitivity. This indicates that the adsorption/chemisorption of oxygen or surface lattice oxygen atoms plays a dominant role in the complete oxidation of molecules. These findings highlight the impact of SnO_2_ addition on the structural and gas-sensing properties of Mn_1−x_CuFe_2_O_4_ nanocomposites. The changes in grain size, strain value, particle size, and gas sensitivity provide valuable insights into the design and optimization of gas-sensing materials for specific applications.

Wei et al. [254] successfully synthesized MOF-based Fe_2_O_3_/ZnFe_2_O_4_ porous nanocomposites using a solvothermal method. The nanocomposites consist of spindles-like Fe_2_O_3_ with a length of about 2 μm and a width of about 400 nm (Figure 10d), which are uniformly adhered to ZnFe_2_O_4_ nanoparticles. Through the analysis of the TEA (triethylamine) gas-sensing mechanism, it was observed that the heterojunction between the spindles-like Fe_2_O_3_ and ZnFe_2_O_4_ nanoparticles played a crucial role in improving the gas-sensing performance. Compared with pure MOF-derived Fe_2_O_3_ spindles, the gas-sensitive properties of Fe_2_O_3_/ZnFe_2_O_4_ nanocomposites were enhanced and exhibited a remarkable response value of up to 69.24 when exposed to 100 ppm TEA (Figure 10e). This indicates a significant improvement in the gas-sensing performance of the nanocomposites compared with the pure Fe_2_O_3_ spindles derived from MOF. Using Cu@carbon as a sacrificial template, Li et al. [29] successfully synthesized CuFe_2_O_4_/α-Fe_2_O_3_ hollow spheres with a diameter of ~210 nm with porous non-thin shells (Figure 10h) by thermal oxidation and solid phase reaction. The gas-sensitive properties of CuFe_2_O_4_/α-Fe_2_O_3_ composites were compared with those of pure α-Fe_2_O_3_ hollow spheres. As anticipated, the sensor based on the CuFe_2_O_4_/α-Fe_2_O_3_ composite exhibited a higher sensitivity (Ra/Rg = 14), faster response and recovery times (6 s/100 s), and lower detection limits (100 ppb) compared with the original α-Fe_2_O_3_ hollow spheres (Figure 10i). The enhanced sensing performance of the CuFe_2_O_4_/α-Fe_2_O_3_ composites can be attributed to several factors. Firstly, the hollow porous structure of the composites provides a larger surface area, which increases the number of active sites for gas adsorption and improves sensitivity. Additionally, the presence of the heterojunction between CuFe_2_O_4_ and α-Fe_2_O_3_ allows for modulation of the resistance and facilitates charge transfer, further enhancing the gas-sensing performance. Lastly, the catalytic performance of CuFe_2_O_4_ in the composites contributes to the improved sensing properties.

**Figure 11:**
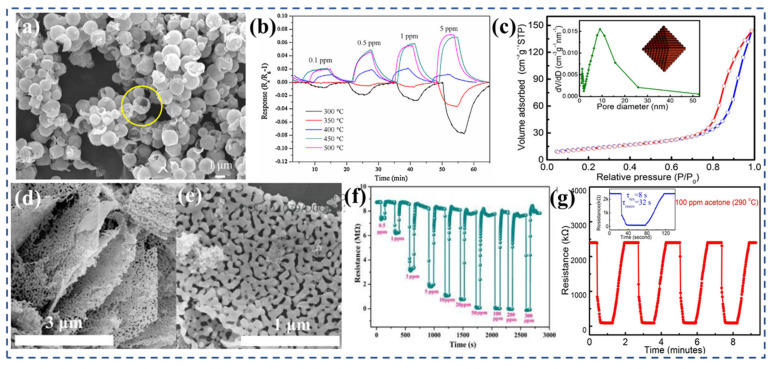
(**a**) SEM images of hollow ZnO/ZnFe_2_O_4_ microspheres. (**b**) Response value towards acetone with 0.1–5 ppm. (**c**) N_2_ adsorption−desorption isotherms for ZnO/ZnFe_2_O_4_ nanocages (inset is the pore size distribution). (**d**,**e**) SEM images of the coral-like ZnO/ZnFe_2_O_4_ with different magnifications. (**f**) Dynamic response/recover curves of the coral-like ZnO/ZnFe_2_O_4_ to different TEA concentrations at 240 °C. (**g**) Dynamic continuous response of ZnO/ZnFe_2_O_4_ hollow nanocages to 100 ppm acetone at 290 °C. (**a**,**b**) Reproduced with permission [287], copyright 2017, Elsevier B.V. (**c**,**g**) Reproduced with permission [278], copyright 2017, Elsevier B.V. (**d**–**f**) Reproduced with permission [41], copyright 2019, Elsevier B.V.

Li et al. [287] utilized a metal–organic skeleton to prepare a precursor similar to Prussian blue, and then employed direct pyrolysis to fabricate hollow ZnO/ZnFe_2_O_4_ microspheres with a heterogeneous structure (Figure 11a). These microspheres had a diameter of approximately 1.5 μm. As a gas-sensitive material, the hollow ZnO/ZnFe_2_O_4_ microspheres exhibited a temperature-dependent n–p–n-type abnormal conductive transition (Figure 11b) when detecting low concentrations of volatile organic compounds (VOCs) such as ethanol, acetone, toluene, and benzene. This phenomenon can be primarily attributed to the interplay of highly separated electron–hole pairs caused by the staggered band arrangement at the heterogeneous interface of the ZnO-ZnFe_2_O_4_ shell. This interplay is influenced by the heat-dependent ionization reaction of the surface-absorbed oxygen molecules and the additional electron injection resulting from the reducing VOCs’ surface reaction during the gas-sensitive process. The abnormal conductive transition observed in the hollow ZnO/ZnFe_2_O_4_ microspheres when exposed to low concentrations of VOCs is a result of the complex interplay between the different processes occurring at the heterogeneous interface. This understanding of the underlying mechanism contributes to the understanding and optimization of gas-sensing properties for applications in VOC detection. Wang et al. [278] devised a design and synthesis method to create ZnO/ZnFe_2_O_4_ hollow nanocages with a diameter of around 100 nm using a metal–organic framework (MOF) technique. The synthesis process involved two steps: the preparation of Fe(III)MOF-5 nanocages as a precursor, followed by the conversion into ZnO/ZnFe_2_O_4_ hollow nanocages through hot annealing in air. Based on the BET analysis, it is observed that the ZnO/ZnFe_2_O_4_ nanocages, in their as-prepared state, possessed a BET specific surface area of 48.4 m^2^·g^−1^ and an average pore size of 9.1 nm, as determined using the BJH method (Figure 11c). Gas-sensing experiments revealed that the ZnO/ZnFe_2_O_4_ hollow nanocages exhibited a superior response value of 25.8 to 100 ppm acetone (Figure 11g), with a detection limit of 1 ppm at the optimized temperature of 290 °C. This response value surpassed that of ZnO hollow nanocages (7.9) and ZnFe_2_O_4_ nanospheres (8.1). Furthermore, the gas-sensing response of the ZnO/ZnFe_2_O_4_ nanocages outperformed that of the other structures, with the response order being as follows: hollow nanocages > double shell > hollow microsphere; hybrid hollow spheres > nanoparticles with rods. Yang et al. [41] conducted a study in which they synthesized coral-like ZnFe_2_O_4_-ZnO heterostructures with mesoporous structures (Figure 11d,e) and evaluated their gas-sensing performance towards the volatile organic compound TEA. The prepared sensor was subjected to thorough gas-sensing tests, and the results demonstrated several advantages, including a high response value (Ra/Rg = 21.3 at 240 °C), fast response and recovery times (0.9 s/23 s), and good repeatability (Figure 11f). The combination of the unique coral-like mesoporous morphology, the formation of n–n heterojunctions, and the synergistic effect of ZnFe_2_O_4′_s Bronsted centers contributed to the improved TEA sensing properties of the coral-like ZnFe_2_O_4_-ZnO. These findings provide valuable insights for the design and optimization of gas-sensing materials for the detection of volatile organic compounds.

### 5.2. Nanostructure Materials/Ferrite

In order to maintain the structural stability of nanostructured materials during heterojunction formation, a two-step method is typically employed [300]. This approach not only maintains the stability of the structural materials, but also suppresses the aggregation of the perovskite iron oxides during synthesis [304].

Nanostructured materials, such as two-dimensional (2D), one-dimensional (1D), and zero-dimensional (0D) structures, possess unique dimensional characteristics that contribute to their attractive physicochemical properties. These structures exhibit small volume, high electron mobility, and large specific surface areas, making them highly advantageous in various applications. In the field of gas sensing, nanostructures with a large surface area and high porosity have been found to significantly enhance the performance of gas sensors. The increased surface area and porosity provide more reaction sites, enabling more efficient interaction between the sensing material and the target gas molecules. This enhanced interaction leads to improved sensitivity and selectivity in gas-sensing applications. A notable strategy to achieve synergistic effects is the integration of metal oxide semiconductor (MOS) materials with nanostructured materials possessing large specific surface areas.

**Figure 12:**
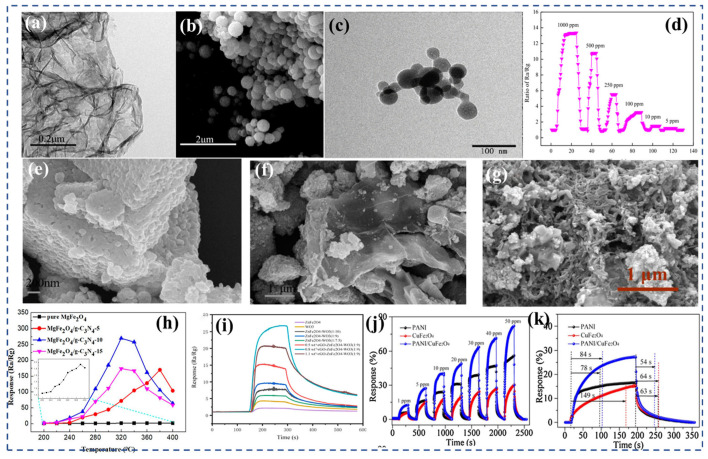
(**a**) TEM image of g-C_3_N_4_. (**b**) SEM image of MgFe_2_O_4_/g-C_3_N_4_ composites. (**c**) TEM images of ZnFe_2_O_4_/GQDs. (**d**) The dynamic response/recover curves of the sensor based on the sample S-15 composite to various concentrations of acetone at RT. (**e**) SEM image of 9WO_3_-ZnFe_2_O_4_. (**f**) SEM micrographs of the 0.8 wt.% rGO/9WO_3_/ZnFe_2_O_4_ composite. (**g**) SEM images of PANI/CuFe_2_O_4_. (**h**) The sensitivity–temperature characteristics of the sensors based on MgFe_2_O_4_/g-C_3_N_4_ composites to 500 ppm acetone. (**i**) Dynamic responses curve of the different ratio of rGO/WO_3_/ZnFe_2_O_4_ composites; (**j**) Response of the sensors toward NH_3_ at 20 °C. (**k**) Response and recovery curves of the sensors toward 5 ppm NH_3_ at 20 °C. (**a**,**b**,**h**) Reproduced with permission [290], copyright 2018, MDPI. (**c**,**d**) Reproduced with permission [291], copyright 2019, Elsevier B.V. (**e**,**f**,**i**) Reproduced with permission [298], copyright 2021, Elsevier B.V. (**g**,**j**,**k**) Reproduced with permission [307], copyright 2020, Elsevier B.V.

Zhang et al. [290] achieved the successful synthesis of porous microsphere composites by incorporating g-C_3_N_4_ into MgFe_2_O_4_ (Figure 12a,b) using a solvothermal method. In the study, the content of g-C_3_N_4_ was varied, and it was found that the sensor based on the MgFe_2_O_4_/g-C_3_N_4_ composite material exhibited excellent gas-sensing performance. Specifically, when the g-C_3_N_4_ content was 10 wt.%, the sensor showed several desirable characteristics, including high sensitivity and selectivity, fast response and recovery times. Notably, the maximum response to acetone increased by approximately 145 times compared with the sensors without g-C_3_N_4_. Moreover, the optimal temperature for sensing was reduced by 60 °C. Chu et al. [291] conducted a study in which they prepared ZnFe_2_O_4_/graphene quantum dot (GQD) nanocomposites (Figure 12c) using a hydrothermal method. The researchers aimed to investigate the influence of GQD content on the gas-sensitive response and selectivity of the ZnFe_2_O_4_/GQD nanocomposites. The results demonstrated that the sensor based on the ZnFe_2_O_4_/GQD nanocomposites exhibited a response of 13.3 to 1000 ppm acetone and a response of 1.2 to 5 ppm acetone at room temperature (Figure 12d). The response time and recovery time for the detection of acetone r were both less than 12 s. However, it should be noted that the long-term gas-sensitive stability of the ZnFe_2_O_4_/GQD nanocomposites was not satisfactory. This indicates that further research and improvement are needed to address the stability issue and enhance the long-term performance of the nanocomposites in gas-sensing applications. Bai et al. [298] synthesized rGO/WO_3_/ZnFe_2_O_4_ composites (Figure 12e,f) with varying proportions using hydrothermal, chemical water bath, and chemical reduction methods. The gas sensitivity of the synthesized composites was tested, yielding noteworthy results. Among the different compositions tested, the 0.8 wt.% rGO-9WO_3_-ZnFe_2_O_4_ terpolymer exhibited a superior gas-sensing performance. It demonstrated a significantly higher response value of 26.92, which is six times higher than that of pure WO_3_ and thirteen times higher than that of ZnFe_2_O_4_ (Figure 12i). Furthermore, the synthesized gas-sensitive material displayed excellent selectivity, a shorter response time of 51 s, and a lower detection limit of 0.02 ppm. These characteristics indicate the enhanced performance of the composite material in terms of sensitivity, selectivity, and response speed compared with the individual components. The successful synthesis of the rGO/WO_3_/ZnFe_2_O_4_ composites and their improved gas-sensing performance suggest their potential for applications in gas-sensing devices. Further optimization and exploration of the composite composition and structure can enable the development of highly efficient gas sensors for various target gases.

### 5.3. Conducting Polymer/Ferrite

In recent years, the synthesis of conductive polymer magnetic nanocomposites has received much attention from researchers because of its lightweight, low-cost preparation methods, and enhanced magnetoelectric properties. Among conductive polymers, polyaniline (PANI) has emerged as a P-type semiconductor material with an excellent sensing ability. While polyaniline-based ammonia sensors have been widely reported, developing faster, highly sensitive, and fully recyclable greenhouse gas sensors remain a major challenge. In this regard, Wang et al. [307] prepared polyaniline/CuFe_2_O_4_ heterostructures (Figure 12j) through in situ polymerization. In contrast with the polyaniline-based sensor, the polyaniline/CoFe_2_O_4_ composite showed a higher response, with a response of up to 27.37% at 5 ppm NH_3_, surpassing the performance of the original PANI and CuFe_2_O_4_ films by a significant margin. This finding suggests that by combining CuFe_2_O_4_ with polyaniline to form a p–n heterojunction, the gas-sensing performance could be enhanced (Figure 12j,k). The p–n heterojunction formed between CuFe_2_O_4_ and polyaniline is expected to improve the gas-sensing performance of polyaniline-based sensors. The synergies between the two materials allows for increased sensitivity, faster response times, and better recoverability.

## 6. Summary and Prospect

This paper provides an exhaustive review of the advancements in spinel-ferrite-based gas sensors, emphasizing three critical areas: nanostructure, elemental doping, and heterostructure. Spinel ferrite gas sensors have garnered interest due to their broad sensitivity and excellent selectivity to various flammable, explosive, toxic, and harmful gases. The gas-sensing mechanism of these sensors depends on intricate interactions and electron transfer at the gas−solid interface. Consequently, alterations in the microstructure of spinel ferrite nanomaterials, such as grain size, specific surface area, and porosity, can substantially influence the sensor’s gas-sensing performance. Metal element doping in spinel ferrite enhances the specific surface area and provides activation energy, while maintaining the original crystal structure. Moreover, the creation of heterojunctions at the interface between different gas-sensitive materials is pivotal in modulating the sensor response by forming an electron depletion layer. A detailed comparison reveals that refining the microstructure, suitable metal element doping, or employing material composites can lead to a certain level of enhancement in the sensing capabilities of gas sensors based on spinel ferrite. Nonetheless, practical applications face challenges, including high power consumption due to thermal excitation effects and extended recovery times due to slow gas desorption. Therefore, innovative research directions are required to achieve swift sensor recuperation and consistent detection at low temperatures, potentially even at ambient room temperature. To overcome these challenges, we suggest a blend of the aforementioned strategies, which may encompass refining the microstructure of spinel ferrites or controlling the iron stoichiometry, designing composite materials composed of spinel ferrite multi-layer porous shells or hollow spheres integrated with nanostructured materials such as reduced graphene oxide and molybdenum disulfide, and developing multi-component hybrid materials. These strategies aim to boost the performance of spinel ferrite gas sensors, with a primary emphasis on achieving a high response and low operating temperatures.

## Figures and Tables

**Table 1:** Summary of the reported spinel ferrite nanoparticles-based gas sensors.

Materials	Synthesis	Morphology	Gas	O.T. (°C)	Conc. (ppm)	Response	t_res_/t_rec_	LOD	Refs.
CdFe_2_O_4_	Co-precipitating	Nanoparticles	Ethanol	300	1000	48 a	-	-	[43]
CdFe_2_O_4_	Co-precipitating	Nanoparticles	CH_3_SH	300	0.01	2 a	600 s/-	-	[44]
CoFe_2_O_4_	Citrate process	Nanoparticles	H_2_S	225	-	0.6 c	-	-	[45]
CoFe_2_O_4_	Hydrothermal	Nanoparticles	TEA	190	50	4.5 a	100 s/120 s	-	[46]
CoFe_2_O_4_	Hydrothermal	Nanoparticles	Ethanol	150	50	6 a	50 s/60 s	-	[46]
CoFe_2_O_4_	Co-precipitation	Nanoparticles (12 nm)	LPG	RT	5 vol.%	2700 c	30 s/60 s	-	[22]
CoFe_2_O_4_	Sol–gel auto-combustion	Nanoparticles	NH_3_	RT	100	0.7 c	118 s/145 s	-	[47]
CoFe_2_O_4_	Sol–gel method	Nanoparticles (~50 nm)	Ethanol	300	150	0.72 c	75 s/110 s	-	[48]
CoFe_2_O_4_	Microwave-assisted	Nanoparticles	SO_2_	120	3.5	3.50 d	10 s/20 s	250 ppb	[49]
CoFe_2_O_4_	Hydrothermal	Nanoparticles	Ethanol	200	100	110 a	15 s/18 s	-	[35]
CoFe_2_O_4_	Solution phase reaction	hexagonally nanoparticle	CO	400	100	3 a	-	-	[50]
CoFe_2_O_4_	Spray pyrolysis	Nanoparticles (54 nm)	LPG	250	5	0.2 c	-	-	[51]
CoFe_2_O_4_	Uniaxial press	Nanoparticles (5.8 nm)	LPG	250	200	0.72 c	3.8 s/43.2 s	-	[52]
CoFe_2_O_4_	Solvothermal	Nanoparticles (10 nm)	Acetone	220	100	17.3 a	27 s/7 s	-	[53]
CoFe_2_O_4_	Hydrothermal	Nanoparticles	CH_3_OH	90	100	0.42 c	-	-	[54]
CoFe_2_O_4_	Hydrothermal	Porous nanoparticles	CH_3_OH	RT	100	0.20 c	-	-	[54]
CuFe_2_O_4_	Citrate process	Nanoparticles	CO	200	-	0.4 c	-	-	[45]
CuFe_2_O_4_	Solid-state reaction	Nanoparticles (70–150 nm)	Ethanol	332	1000	7.5 a	10 s/15 s	-	[55]
CuFe_2_O_4_	Sol–gel	Nanoparticles	LPG	300	150	0.45 c	180 s/240 s	-	[56]
CuFe_2_O_4_	Co-precipitation	Nanoparticles	LPG	RT	1 vol.%	2.6 c	30 s/200 s	-	[57]
CuFe_2_O_4_	Sol–gel technique	Nanoparticles (35.8 ± 5.3 nm)	H_2_S	80	25	0.15 c	51.5 s/-	-	[58]
CuFe_2_O_4_	Co-precipitation	Nanoparticle (7 ± 2.1 nm)	H_2_S	80	300	0.39 d	21.9 s/-	-	[25]
CuFe_2_O_4_	Sputtering	Nanoparticles	H_2_	50	1 vol.%	0.15 c	48 ± 11 s/-	-	[59]
CuFe_2_O_4_	Co-precipitation and annealed	Nanoparticles (22 ± 3 nm)	H_2_S	140	300	0.3 d	32 ± 10 s/-	-	[60]
CuFe_2_O_4_	Co-precipitation	Nanoparticles (6.4 nm)	NH_3_	RT	20	0.6 c	8 s/300 s	-	[37]
MgFe_2_O_4_	Solid-state reaction	Nanoparticles	H_2_S	160	2	13 b	-	-	[61]
MgFe_2_O_4_	Solid-state reaction	Nanoparticles (15–30 nm)	Ethanol	350	50	13 b	-	-	[61]
MgFe_2_O_4_	Co-precipitation method	Particle (1 µm)	LPG	250	3	3 a	-	-	[62]
MgFe_2_O_4_	Wet chemical	Nanoparticles (84 nm)	H_2_	315	1000	0.53 c	-	-	[63]
MgFe_2_O_4_	Wet chemical	Nanoparticles	H_2_	250	1660	1.02 c	-	-	[64]
MgFe_2_O_4_	Sol–gel	Nanoparticles (38 nm)	LPG	325	2000	0.71 c	-	-	[65]
MgFe_2_O_4_	Citrate gel combustion	Nanoparticles (37 nm)	LPG	400	100	0.22 c	34 s/67 s	-	[66]
MgFe_2_O_4_	Auto-combustion	Spherical particles (15–20 nm)	Ethanol	275	5	0.73 c	-	-	[67]
MgFe_2_O_4_	Spray pyrolysis	Nanoparticles (25–45 nm)	Acetone	323 k	75	193% c	-	-	[68]
MgFe_2_O_4_	Co-precipitation	0.9 ± 0.2 µm	CO_2_	300	5000	0.36 c	120 s/240 s	-	[69]
MgFe_2_O_4_	Sol gel combustion	0.18 ± 0.06 µm	CO_2_	300	5000	0.24 c	300 s/300 s	-	[69]
MgFe_2_O_4_	Sol–gel synthesis	Nanoparticles	LPG	RT	4 vol%	27.9% c	158 s/152 s	-	[70]
MgFe_2_O_4_	Polymerization method	Nanoparticles (120 nm)	NO_2_	300	10	39.5 a	-	-	[71]
MgFe_2_O_4_	Reverse coprecipitation	Nanoparticles (132 nm)	NO_2_	300	10	15 a	-	-	[71]
MgFe_2_O_4_	Auto-combustion	Nanoparticles (41 nm)	LPG	250	5	0.3 c	-	-	[72]
NiFe_2_O_4_	Citrate process	Nanoparticles	Cl_2_	300	-	0.75 c	-	-	[45]
NiFe_2_O_4_	Reverse micelle	Nanoparticles	LPG	380	100	0.18 c	-	-	[73]
NiFe_2_O_4_	Hydrothermal	Nanoparticles	LPG	200	100	0.4 c	-	-	[73]
NiFe_2_O_4_	Pulsed wire discharge	Nanoparticles (18–45 nm)	Cl_2_	350	500	0.39 c	-	-	[74]
NiFe_2_O_4_	Ion beam sputtering	Nanoparticles (35 nm)	CH_4_	130	20,000	1.12 a	-	-	[75]
NiFe_2_O_4_	Sol–gel auto combustion	Nanoparticles	Acetone	275	500	4.65 c	170 s/600 s	-	[76]
NiFe_2_O_4_	Glycine combustion	Nanoparticles (38 nm)	LPG	350	2000	375% a	40 s/140 s	-	[77]
NiFe_2_O_4_	Sol–gel self-combustion	Nanoparticles (5.35 nm)	H_2_S	150	200	0.75 c	60 s/300 s	-	[78]
NiFe_2_O_4_	Sol–gel method	Nanoparticles (23 nm)	LPG	RT	2000	2.1 a	72 s/183 s	-	[79]
NiFe_2_O_4_	Sol–gel method	Nanoparticles (23 nm)	CO_2_	RT	2000	1.3 b	100 s/400 s	-	[79]
NiFe_2_O_4_	Co-precipitation	Nanoparticles (15 nm)	LPG	RT	4 vol.%	62.3 b	200 s/250 s	-	[23]
NiFe_2_O_4_	Auto-combustion	Nanoparticles (17 nm)	NH_3_	400	250	23% c	100 s/119 s	-	[80]
NiFe_2_O_4_	Ligand-assisted self-assembly	Nanoparticles (20–40 nm)	Acetone	210	200	57 a	44 s/24 s	-	[81]
NiFe_2_O_4_	Combustion	Nanoparticles (16 nm)	LPG	300	3000	35.62% c	-	-	[82]
NiFe_2_O_4_	Auto-combustion	Nanoparticles	NH_3_	410K	1000	65.29% c	-	-	[83]
NiFe_2_O_4_	Hydrothermal	Nanoparticles	Acetone	190	100	120% c	70 s/130 s	-	[84]
ZnFe_2_O_4_	Citrate process	Nanoparticles	H_2_S	200	-	0.65 c	-	-	[45]
ZnFe_2_O_4_	W/O microemulsion	Nanoparticles	Cl_2_	270	50	83.6 a	4 s/30 s	-	[85]
ZnFe_2_O_4_	Hydrothermal	Nanoparticles	Ethanol	180	100	76 a	-	-	[86]
ZnFe_2_O_4_	W/O microemulsion	Spherical particles (30 nm)	Cl_2_	270	50	85 a	4 s/30 s	-	[38]
ZnFe_2_O_4_	Solid-state reaction	Nanoparticles (15–20 nm)	H_2_S	250	200	3.25 a	20 s/90 s	-	[87]
ZnFe_2_O_4_	Co-precipitation	Nanoparticles	Acetone	300	1000	100,000 a	-	-	[88]
ZnFe_2_O_4_	Wet chemical	Nanoparticles (25–30 nm)	Ethanol	350	100	0.6 c	-	-	[26]
ZnFe_2_O_4_	Glycine combustion	Nanoparticles (25–30 nm)	Acetone	250	2000	57% c	-	-	[89]
ZnFe_2_O_4_	Co-precipitation	Nanoparticles (65 nm)	Ethanol	190	100	0.82 c	30 s/90 s	-	[90]
ZnFe_2_O_4_	Co-precipitation	Nanoparticles (65 nm)	Cl_2_	152	500	0.75 c	20 s/50 s	-	[90]
ZnFe_2_O_4_	Auto combustion	Spherical particles (10 nm)	Ethanol	250	200	1.35 a	70 s/90 s	-	[91]
ZnFe_2_O_4_	Hydrothermal	Nanoparticles (10 nm)	Acetone	200	200	39.5 a	-	-	[92]
ZnFe_2_O_4_	Solid-state	Nanoparticles	HCHO	260	100	37.3 a	4 s/17 s	-	[93]
ZnFe_2_O_4_	Solid-state	Nanoparticles	Ethanol	300	100	29.1 a	2 s/7 s	-	[93]
ZnFe_2_O_4_	Sol–gel self-combustion	Nanoparticles (7 nm)	H_2_S	150	200	0.82 c	40 s/210 s	-	[94]
ZnFe_2_O_4_	Molten salt route	Nanoparticles (27 nm)	H_2_S	260	50	22.5 a	8 s/20 s	-	[95]
ZnFe_2_O_4_	Co-precipitation	Nanoparticles (5 ± 1.4 nm)	H_2_S	80	300	0.64 d	20.1 s/-	-	[25]
ZnFe_2_O_4_	Plasma spraying	Nanoparticles (30 nm)	Acetone	200	100	2.7 a	-	-	[96]
ZnFe_2_O_4_	Plasma spraying	Nanoparticles (30 nm)	Acetone	200	100	2.7 a	-	1.8 ppm	[97]
ZnFe_2_O_4_	Co-precipitation	Nanoparticles (4.8 nm)	Ethanol	300 k	40	37.1a	50 s/116 s	-	[98]
ZnFe_2_O_4_	Hydrothermal	Nanoparticles (10 nm)	NO_2_	125	10	247.7 b	6.5 s/11 s	-	[99]
ZnFe_2_O_4_	Self-catalyzed treatment	Nanoparticles (20 nm)	Acetone	280	100	27.6 a	6 s/4 s	-	[100]
ZnFe_2_O_4_	Ball milling and annealed	Nanoparticles (23.03 nm)	NO_2_	600	300	5% d	145 s/20 s	-	[42]
ZnFe_2_O_4_	Hydrothermal	Nanoparticles (23 nm)	Ethanol	220	40	202.5 a	56 s/46 s	-	[101]
ZnFe_2_O_4_	Hydrothermal	Nanoparticles	O_3_	200	0.03	3.7 a	-	-	[27]
ZnFe_2_O_4_	Solvothermal	Nanoparticles	H_2_S	250	2	498% d	48 s/74 s	-	[102]
ZnFe_2_O_4_	PLD	Nanoparticles (48 nm)	LPG	375	5000	93% c	110 s/180 s	-	[103]
ZnFe_2_O_4_	Sol–gel	Nanoparticles (100 nm)	Ethanol	350	150	0.37 c	120 s/240 s	-	[56]
ZnFe_2_O_4_	Wet chemical	-	H_2_	350	1000	0.47 c	33 s/199 s	-	[104]
ZnFe_2_O_4_	Solid-phase reaction	Nanoparticles	Ethanol	332	100	21.5 a	4 s/14 s	-	[104]
ZnFe_2_O_4_	Solid-phase	Nanoparticles	H_2_S	240	100	14.8 a	7 s/25 s	-	[105]
ZnFe_2_O_4_	Solid-state reaction	Nanoparticles (37.8 nm)	Humidity	-	-	2895 a	-	-	[106]
ZnFe_2_O_4_	Sol–gel auto combustion	Nanoparticles (20 nm)	Ethanol	275	100	4.1 c	10 s/40 s	-	[107]
ZnFe_2_O_4_	Spray pyrolysis	Nanoparticles (61 nm)	LPG	300	5	0.26 c	-	-	[51]
ZnFe_2_O_4_	Screen printing	Nanoparticles (4 nm)	LPG	RT	5 vol.%	16 a	120 s/150 s	-	[108]
ZnFe_2_O_4_	Sol–gel auto-combustion	Nanoparticles	NH_3_	RT	100	0.81 c	381 s/333 s	-	[109]
ZnFe_2_O_4_	Solvothermal	Nanoparticles (21.6 nm)	H_2_S	135	5	15.1 a	30 s/120 s	-	[110]
ZnFe_2_O_4_	MOF and annealing treatment	Porous olive-shaped nanoparticles	Ethanol	120	200	223 a	10 s/184 s	-	[111]

O.T. operating temperature; Conc. concentration; t_res_/t_rec_ response time/recovery time; LOD limit of detection. a Response is defined as R_a_/R_g_; b Response is defined as R_g_/R_a_; c Response is defined as ∆R/R_a_; d Response is defined as ∆R/R_g_. R_a_: resistance of the sensor in air; R_g_: resistance of the sensor exposed to target gas; ∆R: the change in resistance, which equals |R_a_–R_g_|.

**Table 2:** Summary of the reported spinel ferrite nanorods/nanotubes-based gas sensors.

Materials	Synthesis	Morphology	Gas	O.T. (°C)	Conc. (ppm)	Response	t_res_/t_rec_	LOD	Refs.
CoFe_2_O_4_	Electrospinning	Nanofibers	NH_3_	RT	900	0.42 a	-	25 ppm	[112]
CoFe_2_O_4_	Hydrothermal	Nanorods	CH_3_OH	90	100	13.3% c	-	-	[54]
NiFe_2_O_4_	Hydrothermal	Nanorods	NH_3_	150	100	5 a	-	-	[113]
NiFe_2_O_4_	Solvothermal	Nanorods	LPG	200	200	0.687 c	114/18 s	-	[114]
NiFe_2_O_4_	Annealing treatment	Nanorods	n-propanol	120	100	89.2 a	19/41 s	0.41 ppm	[115]
NiFe_2_O_4_	Hydrothermal	Nanorod	Acetone	310	100	70% c	45/75 s	-	[84]
NiFe_2_O_4_	Hydrothermal	Nanorods	TEA	175	1	7 a	12 s/-	-	[116]
NiFe_2_O_4_	Hydrothermal	Nanorods	Toluene	200	500	59.64 a	-	1 ppm	[117]
ZnFe_2_O_4_	Sol–gel template	Tubes	LPG	300	500	17.56 a	-	-	[118]
ZnFe_2_O_4_	Microemulsion and calcination	Porous nanorods	Ethanol	RT	50	14 a	-	-	[119]
ZnFe_2_O_4_	Sol–gel	Aligned nanorods	LPG	RT	5000	4.35 a	60/220 s	-	[120]
ZnFe_2_O_4_	Sol–gel spin coating	Nanorods	LPG	RT	2000	140% c	-	-	[121]
ZnFe_2_O_4_	Hydrothermal	Nanorods	Acetone	260	100	52.8 a	1 s/11 s	-	[122]
ZnFe_2_O_4_	Electrospinning	Nanofiber	H_2_S	350	1	102 a	-	-	[123]
ZnFe_2_O_4_	Electrospinning	Nanofibers	Acetone	190	1000 μL/L	13.5 a	15/17 s	1 μL/L	[124]
CuFe_2_O_4_	Co-precipitation	Nanorods	LPG	RT	5 vol.%	0.57 c	150/510 s	-	[125]

a Response is defined as R_a_/R_g_; c Response is defined as ∆R/R_a_.

**Table 3:** Summary of the reported spinel ferrite nanosheets -based gas sensors.

Materials	Synthesis	Morphology	Gas	O.T. (°C)	Conc. (ppm)	Response	t_res_/t_rec_	LOD	Refs.
CuFe_2_O_4_	Sol–gel	Porous hierarchical	LPG	RT	5000	96% d	60 s/-	-	[126]
MgFe_2_O_4_	Sol–gel	Thick films	acetone	725 K	1000	80% c	13/6 s	-	[127]
ZnFe_2_O_4_	Spray pyrolysis	Thin film	ethanol	390	5	1.2 c	40/120 s	1 ppm	[128]
ZnFe_2_O_4_	hydrothermal	Porous nanosheets	H_2_S	85	5	123 a	39/34 s	0.5 ppm	[129]
ZnFe_2_O_4_	Sol gel	Thin films	LPG	375	900,000	79% c	110/180 s		[103]
ZnFe_2_O_4_	Spray pyrolysis	Thick films	SO_2_	150	100	25% c	-	-	[130]

a Response is defined as R_a_/R_g_; c Response is defined as ∆R/R_a_; d Response is defined as ∆R/R_g_.

**Table 4:** Summary of the reported spinel ferrite nanosphere-based gas sensors.

Materials	Synthesis	Morphology	Gas	O.T. (°C)	Conc. (ppm)	Response	t_res_/t_rec_	LOD	Refs.
CoFe_2_O_4_	Solvothermal	Nanospheres	n-butanol	300	100	42.3 a	-	-	[131]
CoFe_2_O_4_	Self-templating	Double-shelled spheres	NH_3_	240	20	0.514 c	19.6/12.1 s	-	[132]
CuFe_2_O_4_	Solvothermal and annealing	Hollow microspheres	TEA	105	10	4 b	32 s/192 s	-	[133]
CuFe_2_O_4_	Solvothermal	Porous nanospheres	Acetone	250	100	20.1 a	3 s/185 s	-	[134]
NiFe_2_O_4_	Metal–organic framework (MOF)	Ultrathin framework	Toluene	230	1	77.3 b	-	2 ppb	[135]
NiFe_2_O_4_	Hydrothermal	Octahedral	Acetone	120	100	18.8 a	6 s/13 s	-	[136]
NiFe_2_O_4_	Solvothermal and annealing	Hollow hexagonal biyramids	n-propanol	120	200	32.19 a	-	-	[137]
NiFe_2_O_4_	Metal–organic framework	Hollow microspindles	Acetone	120	200	52.8 a	14.2 s/-	-	[138]
NiFe_2_O_4_	Hydrothermal and Co-precipitation	Core−shell nanosphere	Acetone	280	100	10.6 a	1 s/7 s	-	[139]
NiFe_2_O_4_	Solvothermal	Porous microspheres	Acetone	250	100	27.4 a	2 s/-	200 ppb	[140]
NiFe_2_O_4_	Refluxing and calcination	Hollow Nano-Octahedrons	Toluene	260	100	6.41 a	25 s/40 s	1 ppm	[141]
NiFe_2_O_4_	Hydrothermal	MOFs-derived fusiformis	Xylene	300	500	31.52 a	50.10 s/40.30 s	10 ppm	[39]
NiFe_2_O_4_	MOF	Polyhedrons	TEA	190	50	18.9 a	6s/-	-	[142]
NiFe_2_O_4_	Annealing	Nanosheet-Assembled Fluffy Flowers	Ethanol	120	100	23.2 a	-	-	[143]
NiFe_2_O_4_	Hydrothermal	Core−shell architecture	Toluene	240	100	19.95 a	-	1 ppm	[144]
NiFe_2_O_4_	Metal–organic framework	Nanobox	Ethyl acetate	120	200	64.27 b	23 s/62 s	0.26 ppm	[145]
ZnFe_2_O_4_	Hydrothermal	Hollow spheres	Ethanol	225	1000	42.1 a	10 s/8 s	-	[146]
ZnFe_2_O_4_	Solvothermal	Porous nanospheres	Acetone	200	30	12.4 a	9 s/272 s	-	[147]
ZnFe_2_O_4_	Solvothermal	Yolk–shell microspheres	Acetone	200	50	28.3 a	-	-	[148]
ZnFe_2_O_4_	Solvothermal	Hollow microspheres	Acetone	215	20	11.3 a	10 s/200 s	1 ppm	[149]
ZnFe_2_O_4_	Hydrothermal	Nanoflowers	Acetone	300	2000	36.5 a	-	-	[150]
ZnFe_2_O_4_	Nonaqueous	Nanospheres	Toluene	300	100	9.98 a	18 s/29 s	-	[151]
ZnFe_2_O_4_	Solvothermal	Sphere-like hierarchical architectures	Ethanol	180	10	6.85 a	5.1 s/7.2 s	500 ppb	[152]
ZnFe_2_O_4_	Hydrothermal and thermal	Double-shell microspheres	Acetone	206	20	13.6 a	6 s/10 s	0.13 ppm	[153]
ZnFe_2_O_4_	Hydrothermal and calcination	Hollow spheres	Ethylene glycol	200	100	35.5 a	-	-	[154]

a Response is defined as R_a_/R_g_; b Response is defined as R_g_/R_a_; c Response is defined as ∆R/R_a_.

**Table 5:** Summary of the reported spinel ferrite A-site doping-based gas sensors.

Materials	Synthesis	Morphology	Gas	O.T. (°C)	Conc. (ppm)	Response	t_res_/t_rec_	LOD	Refs.
Mg_0_._9_Sn_0_._1_Fe_2_O_4_	Auto-combustion	Nanoparticle (100 nm)	acetone	380	-	0.83 c	3 min/-	-	[165]
Zn_0_._6_Mn_0_._4_Fe_2_O_4_	Sol–gel citrate	Nanoparticle (30–35 nm)	ethanol	300	200	0.78 c	-	-	[166]
Ni_0_._6_Zn_0_._4_Fe_2_O_4_	Sol–gel	Nanoparticle (28–42 nm)	H_2_S	225	50	0.65 c	-	-	[167]
Ni_0_._4_Zn_0_._6_Fe_2_O_4_	Aerosol pyrolysis	Spherical shape (250–600 nm)	NH_3_	350	12.5	0.55 c	-	-	[168]
Cu_0_._5_Co_0_._5_Fe_2_O_4_	Auto-combustion	Nanoparticle (23–43 nm)	H_2_O	RT	80%	11.7	-	-	[169]
10 wt% Ni and 0.2 wt% Sm doped CoFe_2_O_4_	Sol–gel citrate	Nanoparticle (40 nm)	H_2_S	200	1000	0.78 c	5 s/20 s	-	[162]
Ni_0_._3_Zn_0_._7_Fe_2_O_4_	Sol–gel auto combustion	Nanoparticles	Acetone	275	500	2 c	120 s /300 s	-	[160]
Co_0_._8_Ni_0_._2_Fe_2_O_4_	Solvothermal	Nanoparticles (40–90 nm)	NH_3_	-	4000	2.8 a	-	-	[170]
Mn_0_._2_Ni_0_._8_Fe_2_O_4_	Hydrothermal	Nanoparticle (<100 nm)	H_2_O	RT	1000	0.56 c	110 s /160 s	-	[171]
Mg_0_._5_Zn_0_._5_Fe_2_O_4_	Sol Pechini	Embedded nano-tubes	H_2_	350	1660	0.9 c	-	-	[155]
Mg_0_._5_Zn_0_._5_Fe_2_O_4_	Sol Pechini	Isolated nano-tube	H_2_	350	1660	0.66 d	-	-	[24]
Ni_0_._5_Zn_0_._5_Fe_2_O_4_	Co-precipitation	Nanoparticles	NH_3_	305	200	0.7 c	-	-	[24]
Mg_0_._5_Zn_0_._5_Fe_2_O_4_	Sol–gel auto combustion	Nanoparticles (58 nm)	acetone	325	20	0.32 c	137 s /247 s	-	[172]
Mn-CuFe_2_O_4_	Auto-combustion	Nanoparticles (9–45 nm)	LPG	300	1000	0.27 c	-	-	[173]
Mn-CuFe_2_O_4_	Evaporation	Nanoparticles	LPG	250	1000	0.25 c	40 s/40 s	-	[174]
Zn_0_._8_Cu_0_._2_Fe_2_O_4_	Sol–gel	Nanoparticles (10.4 nm)	LPG	RT	2000	2.5 b	60 s /300 s	-	[161]
Ni_0_._6_Zn_0_._4_Fe_2_O_4_	Co-precipitation	Nanoparticles (55 nm)	Cl_2_	177	500	0.66 c	30 s/60 s	-	[90]
Ni_0_._6_Zn_0_._4_Fe_2_O_4_	Microwave	Nanoparticles (25 nm)	Acetone	250	1000	0.72 c	90 s /720 s	-	[175]
Co_0_._5_Ni_0_._5_Fe_2_O_4_	Co-precipitation	Nanoparticles	CO	350	1000	0.25 c	-	-	[176]
Mn-CoFe_2_O_4_	Auto combustion	Nanoparticles (3 nm)	LPG	300	1000	0.19 c	40 s/50 s	-	[177]
1 wt% Cu:NiFe_2_O_4_	Spray pyrolysis deposition	Nanoparticles (40–46 nm)	Ethanol	325	5	3.2 c	-	-	[178]
BaCa_2_Fe_16_O_27_	Sol–gel	Nanoparticles	Ethanol	300	100	0.53 c	-	-	[179]
Mn–CuFe_2_O_4_	Auto-combustion	Nanoparticles (9 nm)	LPG	300	1000	0.28 c	10–20 s/-	-	[180]
Ni-CdFe_2_O_4_	Sol–gel auto combustion	Grain size (300 nm)	H_2_O	RT	-	0.99 c	30 s/45 s	-	[28]
Ni_0_._8_Co_0_._2_Fe_2_O_4_	Evaporation	Nanoparticles (10 nm)	LPG	250	1000	0.7 d	40 s/60 s	-	[181]
Sn_0_._2_Ni_0_._8_Fe_2_O_4_	Co-precipitation	Nanoparticles (35 nm)	SF_6_	RT	80	0.68 c	-	-	[182]
Li-CuFe_2_O_4_	Co-precipitation	Nanoparticle (<100 nm)	LPG	RT	4 vol%	1.82 b	-	-	[183]
In-CuFe_2_O_4_	Co-precipitation	Nanoparticles	LPG	RT	4 vol%	0.37 c	229 s/-	-	[184]
Ni_0_._1_Co_0_._9_Fe_2_O_4_	Sol–gel auto combustion	Microcubes	Acetone	240	200	1.67 b	-	-	[164]
CoNiFe_2_O_4_	Co-precipitation	Nanoparticles (28 nm)	LPG	50	500	0.66 c	-	-	[185]
Bi-CoFe_2_O_4_	Sol–gel	Nanoparticles (5–90 nm)	NO_2_	230	100	0.19 c	31 s/29 s	25 ppm	[186]
Cu-ZnFe_2_O_4_	Hydrothermal	Spherical nanoparticles (50 nm)	H_2_S	RT	5	37.9 a	10 s /210 s	-	[187]
Ni_0_._33_Co_0_._67_Fe_2_O_4_	Solvothermal	Mesoporous microspheres	Toluene	300	10	35 a	10 s/51 s	-	[188]
Zn_0_._5_Cu_0_._5_Fe_2_O_4_	Sol–gel auto-combustion	Nanoparticles (30–70 nm)	H_2_S	80	1000	0.71 c	170 s/-	-	[189]
Mn_0_._7_Zn_0_._3_Fe_2_O_4_	Co-precipitation	Nanoparticles (5.5–10.5 nm)	LPG	250	1000	1.88 a	40 s/20 s	-	[190]
Sn_0_._2_Cu_0_._8_Fe_2_O_4_	Co-precipitation	Nanoparticles (37 nm)	LPG	RT	2 vol%	0.78 c	32 s /111 s	-	[191]
Co_0_._25_Ba_0_._75_Fe_2_O_4_	Co-precipitation	Nanoparticles (16.5 nm)	NO_2_	RT	220	0.79 c	-	-	[192]
Zn_0_._5_Mg_0_._5_Fe_2_O_4_	Co-precipitation	Nanoparticles (50–150 nm)	H_2_S	400	10	0.11 d	16 s/-	-	[193]
Ni_0_._7_Zn_0_._3_Fe_2_O_4_	Co-precipitation and sintering	Nanoparticles	LPG	200	1000	0.75 c	40 s/30 s	-	[163]
Cu_0_._75_Zn_0_._25_Fe_2_O_4_	Solvothermal	Hollow micro-nanospheres	Acetone	125	0.8	2.37 a	66 s /138 s	-	[40]
(Cu,Zn)Fe_2_O_4_	Solvothermal	Nano-microspheres	TEA	165	50	6.77 a	58 s /136 s	-	[194]
CuZnFe_2_O_4_	Electrospinning	Nanofibers	H_2_	250	500	5.9 a	6 s/75 s	-	[195]
5 wt% Ni-doped MnFe_2_O_4_	Co-precipitation	Nanoparticles (35 nm)	NH_3_	RT	200	0.51 c	17 s/13 s	-	[196]
Cu_0_._1_Zn_0_._9_Fe_2_O_4_	Spray pyrolysis	Thin Films	SO_2_	120	200	0.474 c	-	-	[197]
Co_0_._87_Ni_0_._13_Fe_2_O_4_	Co-precipitation	Nanoparticles	LPG	400	5000	0.97 c	11 s /110 s	-	[198]
Ni_0_._4_Mn_0_._3_Zn_0_._3_ Fe_2_O_4_	Precursor combustion	Thick film	Cl_2_	100	300	2.12 d	10 s/15 s	-	[199]
Sr_0_._2_Ni_0_._8_Fe_2_O_4_	Sol–gel spin coating	Nanoparticles (20–50 nm)	LPG	200	2000	0.28 c	78 s/66 s	-	[200]

a Response is defined as R_a_/R_g_; b Response is defined as R_g_/R_a_; c Response is defined as ∆R/R_a_; d Response is defined as ∆R/R_g_.

**Table 10:** Summary of the reported other MOSs/spinel-ferrite-based gas sensors.

Materials	Syntheis	Morphology	Gas	O.T. (°C)	Conc. (ppm)	Response	t_res_/t_rec_	LOD	Refs.
CdO/Cd_0_._1_Ni_0_._45_Mn_0_._45_Fe_2_O_4_	Co-precipitation	Nanoparticles	DMF	250	200	0.85 d	28 s/41 s	-	[240]
Co_3_O_4_/CoFe_2_O_4_	Metal–organic framework	Double-shelled nanotubes	HCHO	139	10	12.7 b	4 s/9 s	300 ppb	[241]
Co_3_O_4_/CoFe_2_O_4_	Calcination	Core–shell structure	NH_3_	220	100	35 a	15 s/21 s	-	[242]
CuO/CuFe_2_O_4_	Frequency sputtering	Thick film	CO_2_	250	5000	0.17 4 c	-	-	[243]
CuO/CuFe_2_O_4_	Calcination	Core–shell	H_2_S	250	2	10.8 a	-	-	[244]
CuO/CuFe_2_O_4_	Frequency sputtering	Thick film	CO_2_	250	5000	0.40 c	-	-	[245]
CuO/CuFe_2_O_4_	Co-precipitation	Nanopowder	CO_2_	350	5000	0.072 c	-	-	[246]
CuO/CuFe_2_O_4_	Radio-frequency sputtering	Thin films	H_2_	400	500	0.79 c	60 s/-	-	[247]
CuO/CuFe_2_O_4_	Water bath and calcination	Microspheres/ nanoparticles	H_2_S	240	10	22.3 a	31 s/40 s	-	[248]
CuO/ZnFe_2_O_4_	Thermal treatment and solvothermal	Yolk–shell microspheres	Xylene	225	100	24.1 a	4 s/6 s	-	[249]
CuO/ZnFe_2_O_4_	Solvothermal	Porous nanospheres	H_2_S	RT	10	0.75 c	70 s/475 s	0.1 ppm	[250]
Fe_2_O_3_/CuFe_2_O_4_	Template-induced method	Hollow spheres	Acetone	250	100	14 a	6 s/100 s	100 ppb	[29]
Fe_2_O_3_/NiFe_2_O_4_	Metal–organic framework	Nanotubes	Acetone	200	100	23 a	4 s/-	-	[251]
Fe_2_O_3_/ZnFe_2_O_4_	Template-induced method	Porous microrods	TEA	305	100	42.4 a	12 s/26 s	-	[252]
Fe_2_O_3_/ZnFe_2_O_4_	Solvothermal	Core–shell nanorods	TEA	280	100	141 a	13 s/30 s	-	[253]
Fe_2_O_3_/ZnFe_2_O_4_	Solvothermal	Spindle-like	TEA	300	100	69.24 a	2 s/7 s	-	[254]
MgO/MgFe_2_O_4_	Co-precipitation	Thick film	H_2_S	200	3	1086 a	18 s/108 s	-	[255]
MgO/MgFe_2_O_4_/Fe_2_O_3_	Calcination	Core–shell microsphere	H_2_S	250	3	1.32 b	-	-	[256]
Mn_2_O_3_/ZnFe_2_O_4_	Co-precipitation	Nanopowder	Ethanol	325	300	0.76 c	-	-	[257]
NiO/NiFe_2_O_4_	Two-step hydrothermal	Nanotetrahedrons/ nanoparticles	HCHO	240	200	33.3 a	12 s/8 s	200 ppb	[258]
NiO/NiFe_2_O_4_	Solvothermal	Nanosheets/ Nanoparticles	Acetone	280	50	23 a	-	-	[259]
PdO/ZnFe_2_O_4_	Ultrasonic spray pyrolysis	Microporous spheres	Acetone	275	100	18.9 a	5 s/54 s	-	[260]
SiO_2_/In_2_O_3_/CoFe_2_O_4_	Hydrothermal	Microspheres	Acetone	260	100	58 a	1 s/59 s	-	[261]
Sn-doped ZnO/ZnFe_2_O_4_	Heat treatment	Porous heterostructures	TEA	270	10	28.1 a	9 s/7 s	0.2 ppm	[262]
SnO_2_/Mn_0_._5_ Cu_0_._5_Fe_2_O_4_	Co-precipitation	Nanoparticles	CO_2_	RT	saturated	18 c	-	-	[263]
SnO_2_/ZnFe_2_O_4_	Sol–gel	Nanoparticles	Acetone	176	100	14.6 a	17 s/23 s	-	[264]
SnO_2_/ZnFe_2_O_4_	Solvothermal	Nanospheres	Acetone	210	100	120 a	30 s/197 s	0.1 ppm	[265]
Y_2_O_3_/CuFe_2_O_4_	Sol–gel auto-combustion	Nanoparticles	Humity	RT	97%	4895 a	9 s/23 s	-	[266]
ZnO/Fe_2_O_3_/ZnFe_2_O_4_	Solvothermal	Thick film	Acetone	190	150	16.2 a	5 s/29 s	-	[267]
ZnO/ZnFe_2_O_4_	Screen-printing	Thick film	Propanol	RT	8000	3.54 c	40 s/70 s	-	[268]
ZnO/ZnFe_2_O_4_	Screen-printing	Thick film	Propanol	RT	1000	5.2 c	45 s/90 s	-	[269]
ZnO/ZnFe_2_O_4_	Screen-printing	Thick film	Propanol	RT	2000	0.15 c	-	-	[270]
ZnO/ZnFe_2_O_4_	Hydrothermal	Hollow microspheres	n-butanol	-	200	27.7 a	10 s/25 s	-	[271]
ZnO/ZnFe_2_O_4_	Solution reactions	Hollow spheres/ nanosheets	Acetone	250	100	16.8 a	1 s/33 s	-	[272]
ZnO/ZnFe_2_O_4_	Two-step sprayed	Backbones/ nanosheets	Ethanol	275	100	10.5 a	-	-	[273]
ZnO/ZnFe_2_O_4_	Hydrothermal	Rod-like	n-butanol	260	50	13.6 a	12 s/11 s	-	[274]
ZnO/ZnFe_2_O_4_	Calcination	Hexagonal	TEA	80	1000	12.7 a	100 s/-	-	[275]
ZnO/ZnFe_2_O_4_	Hydrothermal	Hollow spheres	Acetone	280	50	5.2 b	7.1 s/10.1 s	-	[276]
ZnO/ZnFe_2_O_4_	Pyrolysis	Hollow cube	Acetone	250	5	9.4 a	5.6/6 min	-	[277]
ZnO/ZnFe_2_O_4_	MOF	Hollow nanocages	Acetone	290	100	25.8 a	8 s/32 s	-	[278]
ZnO/ZnFe_2_O_4_	Solution reaction and Co-precipitation	Actinomorphic flower-like	NO_2_	200	1	58 a	7/15 s	-	[279]
ZnO/ZnFe_2_O_4_	Annealing treatment	Triple-shelled hollow microspheres	acetone	140	200	23.5 a	5.2 s/12.8 s	-	[280]
ZnO/ZnFe_2_O_4_	Co-precipitation	Prussian blue analogue	TEA	170	100	7.6 a	1 s/9 s	-	[281]
ZnO/ZnFe_2_O_4_	Hydrolyzation of MOF-5	Nanoparticles	Acetone	190	100	30.8 a	4.7 s/10.3 s	-	[282]
ZnO/ZnFe_2_O_4_	Solvothermal	Core−shell hollow microsphere	Acetone	280	100	33.6 a	8 s/30 s	-	[283]
ZnO/ZnFe_2_O_4_	Solvothermal	Coral-like mesoporous	TEA	240	50	21.3 a	0.9 s/23 s	-	[41]
ZnO/ZnFe_2_O_4_	Solution reaction	Nanosheets assembled microspheres	TMA	240	100	31.5 a	3.1 s/5.7 s	-	[284]
ZnO/ZnFe_2_O_4_	Calcination	Tetrapods/moss-like	H_2_S	250	2	1.5 a	2 s/9 s	0.6 ppb	[285]
ZnO/ZnFe_2_O_4_	MOF	Kiwifruitt-like	TEA	200	100	40.5 a	32 s/41 s	-	[286]
ZnO/ZnFe_2_O_4_	Pyrolysis	Hollow microspheres	Acetone	200	1	8.7 c	-	-	[287]
ZnO/ZnFe_2_O_4_	Solution and Calcination	Microflowers	Acetone	250	50	8.3 a	2 s/-	-	[288]
ZnO/ZnFe_2_O_4_	Hydrothermal	Nanoparticles	Acetone	120	90	92.9 a	7.7 s/27 s		[36]
ZnO/ZnFe_2_O_4_/Au	Electrospinning, Atomic layer deposition and Solution reaction	Nanomeshes	Acetone	225	100	30.3 a	1 s/-	300 ppb	[219]
ZnO/ZnFe_2_O_4_/Au	Hydrothermal and Co-precipitation	Yolk–shell microspheres assembled from nanosheets	Acetone	206	100	18.18 a	4 s/23 s	0.7 ppm	[222]

a Response is defined as R_a_/R_g_; b Response is defined as R_g_/R_a_; c Response is defined as ∆R/R_a_; d Response is defined as ∆R/R_g_.

**Table 11:** Summary of the reported 2D materials/spinel-ferrite-based gas sensors.

Materials	Synthesis	Morphology	Gas	O.T. (°C)	Conc. (ppm)	Response	t_res_/t_rec_	LOD	Refs.
g-C_3_N_4_/MgFe_2_O_4_	Solvothermal	Nanosheets/ Nanoparticles	Ethanol	300	500	112 a	11 s/46 s	-	[289]
g-C_3_N_4_/MgFe_2_O_4_	Solvothermal	Nanosheets/ Porous microspheres	Acetone	320	500	270 a	49 s/29 s	-	[290]
Graphene quantum dots/ZnFe_2_O_4_	Hydrothermal	Nanoparticles	Acetone	RT	1000	13.3 a	9 s/4 s	-	[291]
Graphene/ZnFe_2_O_4_	Solvothermal	Nanosheets/ Nanoparticles	Acetone	275	1000	9.1 a	0.7 s/24.7 s	-	[292]
MoS_2_/CuFe_2_O_4_	Electrospinning	Nanosheets/ Nanotubes	Acetone	RT	100	16.4 a	-	-	[293]
MWCNTs/NiFe_2_O_4_	Sol–gel	Nanotube/ Nanoparticles	H_2_S	300	100	2.5 a	-	-	[294]
MWCNTs/Co_0_._8_Ni_0_._2_Fe_2_O_4_	Solvothermal	Nanotubes/ Nanoparticles	NH_3_	-	4000	6.2 a	-	-	[295]
rGO/CuFe_2_O_4_	Combustion	Nanosheets/ Nanoparticles	NH_3_	RT	50	0.093 c	3 s/6 s	-	[296]
rGO/NiFe_2_O_4_	Hydrothermal	Nanosheets/ Nanoparticles	H_2_	80	200	3.85 a	32 s/85 s	-	[297]
rGO/WO_3_/ZnFe_2_O_4_	Water bath	Nanosheets/Massive/Nanoparticles	TEA	130	10	26.92 a	51 s/144 s	-	[298]
rGO/ZnFe_2_O_4_	Hydrothermal	Nanosheet/Nanorods	SO_2_	RT	100	0.183 c	46 s/54 s	-	[299]
rGO/ZnFe_2_O_4_	Calcination	Nanosheets/ Hollow spheres	Acetone	200	10	8.18 a	23 s/203 s	0.8 ppm	[300]
rGO/ZnFe_2_O_4_	Electrospinning and Calcination	Nanosheets/ Nanofibers	H_2_S	350	1	147 a	-	-	[301]
rGO/ZnFe_2_O_4_	Solvothermal	Nanosheets/ Nanosheets	Ethanol	210	100	41.5 a	14 s/37 s	-	[302]
rGO/ZnFe_2_O_4_	Chemical precipitation	Nanosheets/ Hollow octahedron	NO_2_	RT	2	1.123 d	50 s/250 s	0.14 ppb	[303]
rGO/ZnFe_2_O_4_/Pd	Microwave	Nanosheets/ Nanoparticles	H_2_	RT	200	0.11 c	18 s/39 s	-	[304]

a Response is defined as R_a_/R_g_; c Response is defined as ∆R/R_a_; d Response is defined as ∆R/R_g_.

**Table 12:** Summary of the reported polymer/spinel-ferrite-based gas sensors.

Materials	Synthesis	Morphology	Gas	O.T. (°C)	Conc. (ppm)	Response	t_res_/t_rec_	LOD	Refs.
Polyacrylic acid/ NiFe_2_O_4_	Solvothermal	Thin film	NH_3_	150	100	4.1 a	-	-	[305]
Polyindole/ ZnFe_2_O_4_	In situ polymerization	Nanosheets/ Nanoparticles	NH_3_	RT	100	0.9 a	-	-	[306]
Polyaniline/ CuFe_2_O_4_	Polymerization	Nanocapsules/ Nanosphere	NH_3_	RT	5	0.27 c	84 s/54 s	-	[307]
Polyaniline/ NiFe_2_O_4_	Electrospinning and polymerization	Nanofibers	NH_3_	RT	100	30.8 c	15 s/21 s	250 ppb	[308]

a Response is defined as R_a_/R_g_; c Response is defined as ∆R/R_a_.

## Data Availability

No new data were created or analyzed in this study. Data sharing is not applicable to this article.
